# The Feasibility and Acceptability of a Remotely Delivered, Combined Exercise Intervention on Cognitive Function in Patients With Breast Cancer Following Chemotherapy: Randomized Controlled Trial

**DOI:** 10.2196/73393

**Published:** 2026-02-23

**Authors:** Linda Trinh, Natalie Cuda, Catherine M Sabiston, Ellen Warner, Jennifer D Ryan, Arthur F Kramer, Michelle Ha, Hui Xiao, Sarah O'Rourke, Edward McAuley

**Affiliations:** 1 Faculty of Kinesiology and Physical Education University of Toronto Toronto, ON Canada; 2 Odette Cancer Centre Sunnybrook Health Science Centre Toronto, ON Canada; 3 Baycrest Academy for Research and Education Rotman Research Institute Toronto, ON Canada; 4 Beckman Institute University of Illinois Urbana, IL United States; 5 Department of Kinesiology and Community Health University of Illinois Urbana-Champaign Urbana, MD United States

**Keywords:** cancer-related cognitive impairment, breast cancer, quality of life, exercise, feasibility study

## Abstract

**Background:**

Cognitive impairments, a prevalent quality-of-life concern in breast cancer (BC), are particularly pronounced in women having undergone adjuvant chemotherapy. These impairments—affecting executive function, attention, and processing speed—are often underdiagnosed, with no established treatments. Exercise is a potential intervention to mitigate cancer-related cognitive impairment (CRCI). Since virtual care delivery is feasible, remotely delivered exercise interventions for CRCI management in patients with BC may be explored.

**Objective:**

We examined the feasibility of an 8-week remotely delivered combined exercise program (aerobic+resistance training) compared to a stretching and toning active control in postchemotherapy patients with BC.

**Methods:**

Patients with BC who completed adjuvant chemotherapy within 48 months were recruited across Canada from February to July 2023. The combined exercise group engaged in unsupervised aerobic exercise (30 minutes, thrice per week), supervised group-based resistance training (30 minutes, twice per week via Zoom [Zoom Communications, Inc]), and one recorded class weekly, supplemented with 4 biweekly behavioral counseling sessions. The active control group participated in low-intensity balance and flexibility classes (30 minutes, twice per week live, once per week recorded). Feasibility was assessed via enrollment, adherence, attrition, measurement completion, adverse events, and participant satisfaction; cognitive function, using the National Institutes of Health Toolbox Cognition Battery Remote Administration (V2) at baseline and post intervention.

**Results:**

Twenty-one participants (mean age 51.6, SD 7.2 years; 11.8, SD 12.9 months since treatment) were randomized to the combined exercise (n=10) or the active control (n=11) group. Final analyses included 18 participants (mean age 51.9, SD 7.4 years; mean months since treatment 12.6, SD 13.5) with 9 participants in each group (51.2% enrollment rate; 14.3% attrition; no adverse events). Measurement completion rates were 85.7% and participants reported high satisfaction with the intervention, indicating minimal burden. Adherence rates for exercise classes were 70.8% in both groups. Adherence to behavioral counseling sessions was 77.5% (combined exercise group only). There were no significant differences in objectively measured cognitive function, but small-to-medium effect size improvements were observed in objectively measured episodic memory (mean difference 5.33, 95% CI –12.5 to 23.2; ηp²=0.03), working memory (mean difference 8.17, 95% CI –4.2 to 20.6; ηp²=0.12), executive function updating (mean difference –394.35, 95% CI –1035.67 to 246.96 ms; ηp²=0.07), and immediate memory and verbal learning (mean difference +3.22, 95% CI –2.0 to 8.5; ηp²=0.12), trending toward the multicomponent exercise group vs the active control group. In contrast, small-to-medium effect size improvements were observed in the Oral Reading Recognition Test (mean difference –9.65, 95% CI –22.9 to 3.5; *P*=.14; ηp²=0.14) and Picture Vocabulary Test (mean difference –2.48, 95% CI –5.2 to 0.3, *P*=.07; ηp²=0.20), trending toward the active control group.

**Conclusions:**

A remotely delivered combined exercise intervention is feasible and may improve CRCI in patients with BC. Larger randomized controlled trials are warranted to confirm its efficacy in enhancing cognitive function and quality of life in this population.

**Trial Registration:**

ClinicalTrials.gov NCT05704855; https://clinicaltrials.gov/study/NCT05704855

## Introduction

An increasing number of patients with breast cancer (BC) are experiencing chronic symptoms due to rising cancer incidence and improving survival rates [1]. Treatments, such as chemotherapy, radiation therapy, and hormone therapy, while effective at reducing mortality, are associated with many adverse side effects, including cognitive decline known as cancer-related cognitive impairment (CRCI) [2]. CRCI has been reported in up to 85% of patients with BC, lasting for months, years, and not uncommonly indefinitely following treatment completion [3]. CRCI manifests as problems with attention, processing speed, memory, and executive function and is most commonly reported following chemotherapy [4-6]. Chemotherapeutic drugs may have neurotoxic effects, leading to structural and functional changes in the brain [4,7-9]. Other chemotherapy-related side effects, such as fatigue, anxiety, depression, stress, and sleep dysfunction, may further degrade cognition in patients with BC [10,11]. These cognitive problems can significantly impair work performance, social relationships, and daily functioning, ultimately reducing quality of life (QoL) [12,13]. Despite the prevalence and significance of this problem, no established treatment has yet been identified to mitigate the adverse effects of CRCI on patients with BC [14].

Although the majority of evidence for CRCI in patients with BC is attributed to chemotherapy, one confounding aspect is the impact of aging and changes in menopausal status that result from chemotherapy-induced menopause and lowered levels of estradiol [15-17]. Case studies in patients with BC show that CRCI may vary in those who received the same course of chemotherapy, potentially due to differences in menopausal status [18]. Schilder et al [19] demonstrated that after one year of adjuvant therapy, tamoxifen use was associated with statistically significant lower functioning in verbal memory and executive functioning, whereas exemestane use was not associated with statistically significant lower cognitive functioning in postmenopausal patients with BC. Klemp et al [20] conducted a longitudinal study examining both subjective and objective measures of cognitive function and QoL in pre- and perimenopausal patients with BC receiving chemotherapy. Assessments were conducted prior to chemotherapy, after cycle 3, within 2-3 weeks of completing adjuvant chemotherapy, and more than 8+ years later. Neither age nor estradiol levels were associated with cognitive complaints. Vega et al [21] compared a group of primarily postmenopausal women with persistent CRCI to 2 groups of postmenopausal women: women who endorse menopause‐associated subjective cognitive decline (maSCD+) and women who do not (maSCD−) to explore the potential role of menopause in CRCI. Women with persistent CRCI reported more severe subjective symptoms of cognitive decline, along with measurable differences in objective performance, compared to women who experienced natural menopause. In addition, women with persistent CRCI reported greater menopausal symptoms compared with the maSCD- group, but not the maSCD+ group. These findings were unrelated to menopausal status prior to chemotherapy or current endocrine therapy use. Although menopausal symptoms may contribute to some CRCI experienced by patients with BC, they do not fully account for CRCI. Future research is needed to determine the specific predictors and causal mechanisms for CRCI.

Implementing combined (aerobic+resistance training) exercise interventions could be a promising strategy to improve cognitive outcomes and reduce the impact of CRCI. Studies with older adults have demonstrated that combined exercise improves memory more than aerobic exercise alone by producing changes in brain structure, brain function, and brain connectivity [22,23]. Recent reviews indicate that exercise interventions, such as aerobic and combined aerobic-resistance training, may help improve cognitive function in patients with BC [3,24]. Across interventions, programs delivered 2-5 times per week for 10-60 minutes, or aerobic training combined with whole-body resistance, most consistently improved short-term self-reported cognition [3,24]. Another scoping review of 97 studies on physical activity (PA) and cognition among cancer survivors, primarily patients with BC, found that 32% of studies reported positive associations, 66% had inconclusive results, and only 2% showed negative associations, with cognition assessed using both objective and subjective measures [25]. While the review encompassed various study designs, including observational and interventional research, most interventions were delivered after, rather than during, chemotherapy. Complementing these findings, the recent ACTIVATE (Aerobic exercise and CogniTIVe functioning in women with breAsT cancEr) trial specifically examined the effects of aerobic exercise during chemotherapy, demonstrating significant improvements in self-reported cognitive function and QoL in women with BC, despite no significant effects on objective cognitive outcomes [26].

Despite growing interest in this area, findings remain preliminary with limited evidence derived from high-quality, adequately powered randomized controlled trials (RCTs) in patients with BC [3,24]. For example, a 3-arm RCT compared 16 weeks of concurrent aerobic-resistance exercise, continuous moderate-intensity aerobic exercise, and high-intensity interval training to usual care in 206 patients with BC following chemotherapy [27]. Immediately post intervention, the aerobic-resistance training group had no changes in self-reported cognitive function [27]. However, lower levels of cognitive cancer-related fatigue were noted at the 2-year follow-up in this group [28]. Nevertheless, the lack of data on the specific type and frequency of exercise during the follow-up period makes it challenging to attribute these improvements solely to the initial aerobic-resistance exercise intervention.

Meanwhile, many trials are limited to subjective measures of cognitive function and do not prioritize objective cognitive function as a primary outcome. While self-reported CRCI is correlated with mood and fatigue, the gold standard for measuring cognitive function, as developed by the International Cognition and Cancer Task Force (ICCTF), is objective tests of processing speed, memory, and executive function [6]. In practice, objective batteries are often relegated to secondary end points, and when included, they tend to show smaller or inconsistent effects compared with perceived cognition. For example, the recent ACTIVATE trial found improvements in self-reported cognitive function and QoL without parallel benefits on objective tests [26]. Similarly, Galiano-Castillo et al [29] conducted an RCT to test the effects of an 8-week remotely delivered combined exercise program on cognitive function for patients with BC. Following this program, improvements were found in short-term memory, measured by the recall of auditory consonant trigrams, but not mental flexibility, measured by Trail Making Test performance. While this study highlights the potential benefits of a remotely delivered combined exercise program on cognitive function, its primary focus was on physical functional capacity, with cognitive function evaluated as a secondary outcome [29]. Koevoets et al [30] examined the effects of a 6-month supervised combined exercise intervention on cognitive function for patients with BC exposed to chemotherapy. Beneficial intervention effects were found for secondary outcomes of self-reported cognitive function, fatigue, QoL, and depression, but not for the primary outcome of total recall measured by the Hopkins Verbal Learning Test-Revised, or other objective measures of cognitive function measured by the Amsterdam Cognition Scan [30].

Overall, key gaps were noted in the literature regarding exercise interventions, including limited understanding of the exercise dose needed to elicit cognitive benefits, insufficient attention to behavior change support required for maintaining long-term exercise engagement and reducing long-term CRCI [3,25,31,32], and a lack of remotely delivered exercise interventions. Moreover, inconsistent inclusion of patients with BC with self-reported CRCI following chemotherapy, absence of active comparators, and considerable heterogeneity in cognitive function measures are also notable gaps in the literature. Attention-matched or active comparators (eg, stretching and toning) are needed to isolate exercise effects and minimize bias [33-35]. Active control groups (receiving intervention during or after the intervention period) have lower contamination (ie, increasing exercise) and maintains participant engagement regardless of group assignment. This noncompliance may lead to decreased power to detect a significant intervention effect [34]. As such, the available evidence is insufficient for expert consensus to guide exercise recommendations for CRCI management and additional research is warranted.

Previous studies were conducted in person under supervised conditions, and little is known about remotely delivered combined exercise interventions. Patients with BC commonly report issues with transportation to fitness centers, weather constraints, and lack of accessibility as barriers to in person exercise [36]. Remotely delivered combined exercise programs have the potential to reduce these barriers and enhance reach and accessibility to patients with BC [29,37]. Preliminary evidence suggests that 10- to 16-week [28,38] supervised or 8-week [29] home-based combined exercise can improve self-reported cognitive function in patients with BC. Systematic reviews have also shown the effectiveness of remotely delivered exercise interventions in improving physical outcomes [39,40], with high adherence and satisfaction rates among patients with BC [41]. Therefore, there is a clear need for more rigorous neuropsychological testing to examine the effects of combined exercise on cognitive function in patients with BC, especially given the promising preliminary evidence supporting its benefits and the limited knowledge of remotely delivered interventions [28,29,38].

The primary purpose of this study was to assess the feasibility of an 8-week, remotely delivered, combined exercise intervention (aerobic+resistance training) on cognitive function in patients with BC following chemotherapy. Feasibility indicators included enrollment rates, adherence (defined as maintaining an exercise rate of ≥70%), attrition rates (<30%), adverse events, participant satisfaction, and feedback. It was hypothesized that the trial would meet the following a priori feasibility indicators, including an adherence rate 70%, an attrition rate <30% [42-44], and that no adverse events or major injuries would occur as a result of the combined exercise intervention. We also predicted that the combined exercise group would result in significantly greater improvements in objective measures of attention, executive function, and memory (secondary outcomes) compared to the active control group at post intervention (8 weeks), with sustained exercise.

## Methods

### Study Design

This 2-armed, parallel-design pilot RCT evaluated the effects of an 8-week, remotely delivered combined (aerobic + resistance training) exercise program compared to an active control group. Participants were randomized in a 1:1 allocation ratio. Reporting of the trial outcomes followed the CONSORT-eHEALTH (Consolidated Standards of Reporting Trials of Electronic and Mobile Health Applications and Online Telehealth; Multimedia Appendix 1) 2011 guidelines [45,46].

### Participants

Participants were recruited from cancer care organizations, support groups, university listserves, an existing database of research individuals who participated in prior research studies in the laboratory, and social media advertisements (ie, Facebook [Meta Platforms, Inc], Instagram [Meta Platforms, Inc], and X [X Corp]) across Canada between February and July 2023. Eligibility criteria included (1) 40-65 years of age, (2) a history of stage I-III BC (nonmetastatic), (3) a history of receiving chemotherapy within 48 months prior to enrollment, (4) mild cognitive impairment as determined by the Modified Telephone Interview of Cognitive Status (TICS-M) [scores between 21 and 24 to separate individuals with mild cognitive impairment and normal cognition (>24)] [47], (5) self-reported low-activity, defined as <3 days of exercise (<20 min/d) per week in the previous 6 months [48], (6) physician clearance to participate in exercise if required, (7) no previous invasive cancer, (8) no neurological or musculoskeletal comorbidity inhibiting exercise, (9) access to a webcam and internet for videoconferencing, and (10) fluency in English. Participants who were interested in the study contacted the study team to schedule a screening call to further assess their eligibility. Additionally, participants were asked to provide their tentative availability for attending 2 live, remotely delivered classes each week.

### Randomization and Blinding

Eligible participants were randomized in a 1:1 ratio to either the combined exercise group or the active control group using REDCap (Research Electronic Data Capture; Vanderbilt University) [49,50] by a graduate research assistant (NC). Randomization was conducted upon completion of the informed consent form, all baseline measures (ie, PA) assessment, and a battery of questionnaires. Participants were blinded to the study hypotheses and were only informed of their assigned group. Outcome assessors were kept blinded to the allocation.

### Intervention

**Figure 1:**
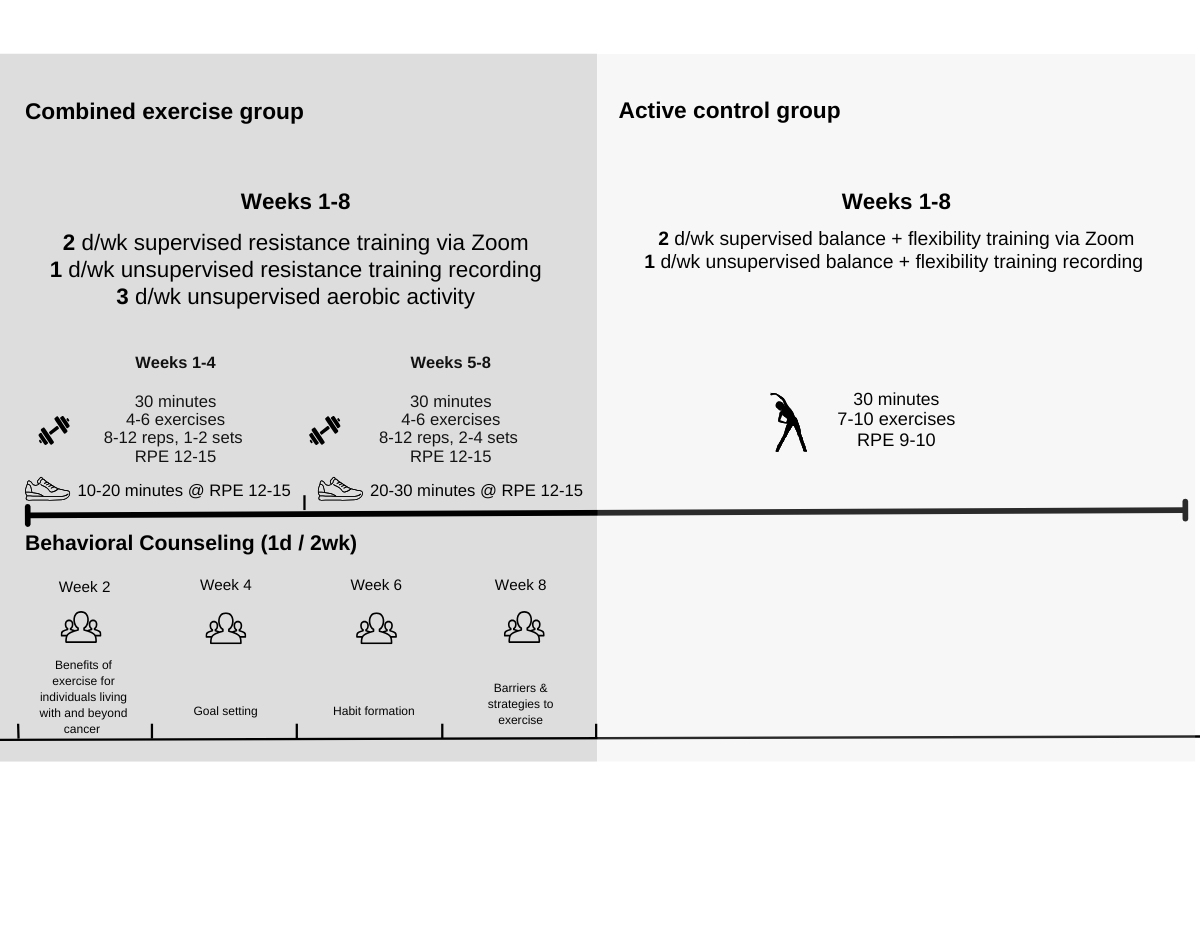
Combined exercise group.

Participants in both groups participated in an 8-week, remotely delivered, supervised exercise program conducted via videoconferencing (Zoom [Zoom Communications, Inc]). The program consisted of two 30-minute live sessions and one 30-minute recorded session per week (Figure 1). Recorded sessions aligned with live classes to maintain consistency and allowed for individualized adjustments. All live sessions were led by Qualified Exercise Professionals (QEPs) certified as Registered Kinesiologists or through the American College of Sports Medicine (ACSM) or Canadian Society of Exercise Physiologists (CSEP). Participants were encouraged to keep their cameras on during live sessions to facilitate safety monitoring and provide real-time exercise modifications.

Participants received resistance bands, a Fitbit Inspire 2 heart rate (HR) monitor, and an exercise log. The program included 30 minutes of unsupervised aerobic exercise (eg, walking) 3 times per week, gradually increasing intensity and duration from 40% to 59% of the maximum HR reserve initially to 60%-70% by the end of the program. The program also included 30-minute supervised resistance training sessions 2 times per week. Resistance training focused on major muscle groups using 2-3 sets of 8-12 repetitions at a Rating of Perceived Exertion (RPE) of 12-15, progressively adjusted with stronger resistance bands. A 30-60 second rest was provided between sets and 60 seconds between exercises, during which the QEP demonstrated the next exercise and offered modifications. Participants recorded their HR and RPE in exercise logs after each session. Participants also attended 4 remotely delivered, biweekly, 30-minute behavioral counseling sessions based on the multi-process action control (M-PAC) framework, emphasizing goal-setting, habit formation, and behavioral control to support long-term PA (Table 1 [51-54]).

**Table 1:** Overview of biweekly behavioral counseling sessions in the combined exercise group.

Week	Topic	Description
2	Benefits of exercise for individuals living with and beyond cancer	Participants learned about the exercise guidelines for general health benefits and the cancer-specific exercise guidelines. Participants were invited to share their meaning of the term “exercise” and some of the exercises they have engaged in or would like to engage in.
4	Goal setting	Participants learned about the importance of goal setting, the difference between short- and long-term goals, SMART^a^ goals, and intrinsic and extrinsic rewards. Participants created and discussed their short- and long-term SMART goals and their prospective intrinsic and extrinsic rewards. Participants were invited to share their responses with the group.
6	Habit formation	Participants learned about habits and how to develop PA^b^ habits, including “tagging” activities to existing environmental cues and creating “cues to action.” Participants brainstormed ideas for creating PA habits using environmental cues that exist within their work and nonworkdays. Participants were invited to share their responses with the group.
8	Barriers and strategies to exercise	Participants learned about cancer-specific barriers and facilitators to exercise. In addition, participants were provided with solutions that may help them overcome some of the barriers to PA. Participants were reminded of past behavioral counseling sessions (ie, revisiting SMART goals and habit formation). Participants applied these concepts into practice by creating a new SMART goal and reward, identifying an environmental cue that will trigger the behavior, and a strategy to overcome any barriers that may arise (or arose) that may get in the way of completing this goal. Participants were invited to share their responses with the group.

^a^SMART: specific, measurable, achievable, relevant, and time-bound.

^b^PA: physical activity.

### Active Control Group

The active control group participated in a low-intensity, whole-body stretching and toning program designed to improve balance and flexibility. Exercises targeted major muscle groups potentially affected by cancer treatments, such as steroid use, radiation therapy, or surgery [55]. Flexibility exercises, incorporating both static and dynamic movements, were introduced weekly to maintain engagement and progress using resistance bands. Intensity was maintained at a light level (RPE 9-10) [56]. Participants recorded their HR (using Fitbit devices) and RPE in exercise logs after each session.

### Measures

#### Primary Outcome: Feasibility

Feasibility was assessed by evaluating the enrollment, adherence (ie, attendance and exercise prescription adherence), attrition rates, measurement completion rates, adverse events, program satisfaction, and therapeutic alliance with the QEP. The enrollment rate was determined by the percentage of participants assessed for eligibility who subsequently enrolled. Attendance was expressed as a percentage of exercise sessions attended. Exercise prescription adherence was determined by assessing self-reported RPE and Fitbit Inspire 2-measured HR during each exercise session. Attrition was measured as the percentage of participants who did not complete the intervention. Participants completed a patient satisfaction and therapeutic alliance questionnaire following the 8-week intervention. The Working Alliance Inventory Short Revised (WAI-SR) [57,58] was used to assess therapeutic alliance, with higher scores representing better therapeutic alliance. Closed-ended questions with response options on a 7-point Likert scale ranging from 1 (not at all) to 7 (very much) were used.

#### Secondary Outcomes

##### Objectively Measured Cognitive Outcomes

Cognitive function was assessed at baseline and post intervention using the remotely administered National Institutes of Health (NIH) Toolbox Cognition Battery (v2), a validated tool with minimal practice effects comparable to other widely used gold-standard cognitive measures [59,60]. The NIH Toolbox Cognition Battery included 4 tests: the Picture Vocabulary Test, which assessed language and vocabulary knowledge; the Picture Sequence Memory Test, which measured episodic memory; the List Sort Working Memory Test, which evaluated working memory and executive function updating; and the Auditory Verbal Learning Test, which assessed immediate memory and verbal learning [59,60]. A supplementary measure of executive function shifting—a domain frequently impaired in BC survivors treated with chemotherapy [14,61]—was measured using the PsyToolkit software platform (Gijsbert Stoet) to administer Task Switching cognitive test [62,63].

All cognitive assessments were administered remotely via Zoom at both time points through a shared iPad (9.7” iPad 2; Apple) screen in a quiet, distraction-free room, with each session lasting about 45 minutes. The examiner completed the remote administration training protocol [64] under the guidance of researchers with expertise in cognitive psychology (AFK and JDR). To ensure standardized administration across participants, a single examiner conducted all assessments through screen-sharing to monitor participants and their responses in real-time.

Device-based exercise minutes were measured at baseline and post intervention with ActiGraph GTX3+ accelerometers. Participants wore the accelerometer on their nondominant hip during waking hours for 7 consecutive days. Data were analyzed if there were no extreme counts (> 20,000) and if participants had at least 10 valid hours of wear time on 4 or more days. Data were downloaded in 60-second epochs, processed, and converted to mean counts per minute in the ActiLife software package (v6.13.5; ActiGraph). These counts were used to estimate daily minutes of light (101-1951 counts min^−1^), moderate (1952-5724 counts min^−1^), vigorous (≥5725 counts min^−1^), and total moderate-to-vigorous intensity exercise (≥1952 counts min^−1^) based on established cut points [65]. Further, weekly minutes spent in each exercise intensity category were calculated and presented.

##### Demographic and Clinical Information

Demographic and clinical information were collected at baseline using a self-reported standardized health history questionnaire used in previous studies [44,66,67]. Demographic variables included age, marital status, education, employment, ethnicity, smoking, and alcohol use. Clinical information included months since diagnosis, months since treatment, type of cancer treatment received, current cancer status, and general health status. Additional data on comorbidities, such as high blood pressure, high cholesterol, diabetes, and arthritis, were also obtained.

### Data Analyses

Data analyses were performed using IBM SPSS Statistics (version 29). As this was a feasibility study, an a priori power calculation was not performed [68,69]. Descriptive statistics (ie, means and SDs) characterized the sample for demographic, clinical, and feasibility outcomes. z-scores were used to identify any outliers in the field. Analyses of covariance (ANCOVA) were used to assess both primary and secondary outcomes of the study. The dependent variable was the mean difference of each intervention outcome (ie, the difference between post- and preintervention scores), while the independent variable was the group (ie, combined exercise or active control). All analyses were conducted while controlling for the baseline values of each outcome. All analyses were conducted on an intention-to-treat basis. Given that the purpose of this feasibility trial was to inform a larger RCT, outcomes were interpreted for potential clinical significance based on the direction and magnitude of numerical differences. Partial eta squared (ηp^2^) values were reported to describe the observed effect sizes.

### Ethical Considerations

The trial protocol was approved by the Research Ethics Board at the University of Toronto (number 43675), and all participants provided written informed consent. Participant privacy and confidentiality were strictly maintained throughout the study; all data were deidentified and stored on secure, password-protected servers accessible only to authorized research personnel, in accordance with institutional and ethical guidelines. Participants received a total of $20 CAD compensation, plus a Fitbit Inspire 2 for their time and participation, consistent with the approved ethics protocol.

## Results

### Sample Characteristics

Demographic and clinical characteristics of the participants are summarized in Table 2. Overall, all participants were women (21/21, 100%), predominantly White (16/21, 76%), with an average age of 51.9 (SD 7.2) years. The majority were married (17/21, 81%), some had completed university or college (10/21, 48%), and many were employed full-time (13/21, 62%). All participants had chemotherapy and were, on average, 22.9 (SD 13.9) months post diagnosis and 11.8 (SD 12.9) months post treatment. Regarding weekly exercise minutes, participants engaged in 149.4 (SD 96.1) moderate-to-vigorous intensity exercise minutes per week.

**Table 2:** Demographic and clinical characteristics of patients with breast cancer at baseline (N=21).

Variable			Value
Age (years), mean (SD)			51.9 (7.2)
**Sex, n (%)**			
		Female	21 (100)
**Marital status,** **n (%)**			
		Married	17 (81)
		Divorced	1 (4.8)
		Common-law	3 (14.3)
**Education,** **n (%)**			
		Completed high school	2 (9.5)
		Some university or college	3 (14.3)
		Completed university or college	10 (47.6)
		Completed graduate school	6 (28.6)
**Employment,** **n (%)**			
		Disability	4 (19)
		Retired	3 (14.3)
		Part-time	1 (4.8)
		Full-time	13 (61.9)
**Race,** **n (%)**			
		White	16 (76.2)
		Chinese	2 (9.5)
		South Asian (East Indian, Pakistani)	2 (9.5)
		Other (did not specify)	1 (4.8)
**Months (n=19),** **mean (SD)**			
		Since treatment	11.8 (12.9)
		Since diagnosis	22.9 (13.8)
**Treatment type,** **n (%)**			
		Chemotherapy	21 (100)
**Current cancer status,** **n (%)**			
		Currently receiving maintenance therapy	10 (47.6)
		Not currently receiving any treatment	9 (42.9)
		Currently receiving primary cancer treatment	2 (9.5)
**General health status,** **n (%)**			
		Good	10 (47.6)
		Fair	6 (28.6)
		Very good	4 (19)
		Excellent	1 (4.8)
**Other comorbidities,** **n (%)**			
		High blood pressure	3 (14.3)
		High cholesterol	3 (14.3)
		Diabetes	1 (4.8)
		Arthritis	2 (9.5)
**Smoking status,** **n (%)**			
	Never smoked		12 (57.1)
	Ex-smoker		8 (38.1)
	Occasional		1 (4.8)
**Alcohol consumption,** **n (%)**			
	Never		7 (33.3)
	Less than once a month		11 (52.4)
	2-3 times per month		2 (9.5)
	2-3 times per week		1 (4.8)
**Exercise minutes (min/wk), mean (SD)**			
	Sedentary		5212. 3 (1033.8)
	Light intensity		2093.6 (487.1)
	Moderate intensity		139.7 (82.9)
	Vigorous intensity		8.4 (26.3)
	Total moderate-to-vigorous intensity		149.4 (96.1)

### Feasibility Outcomes

**Figure 2:**
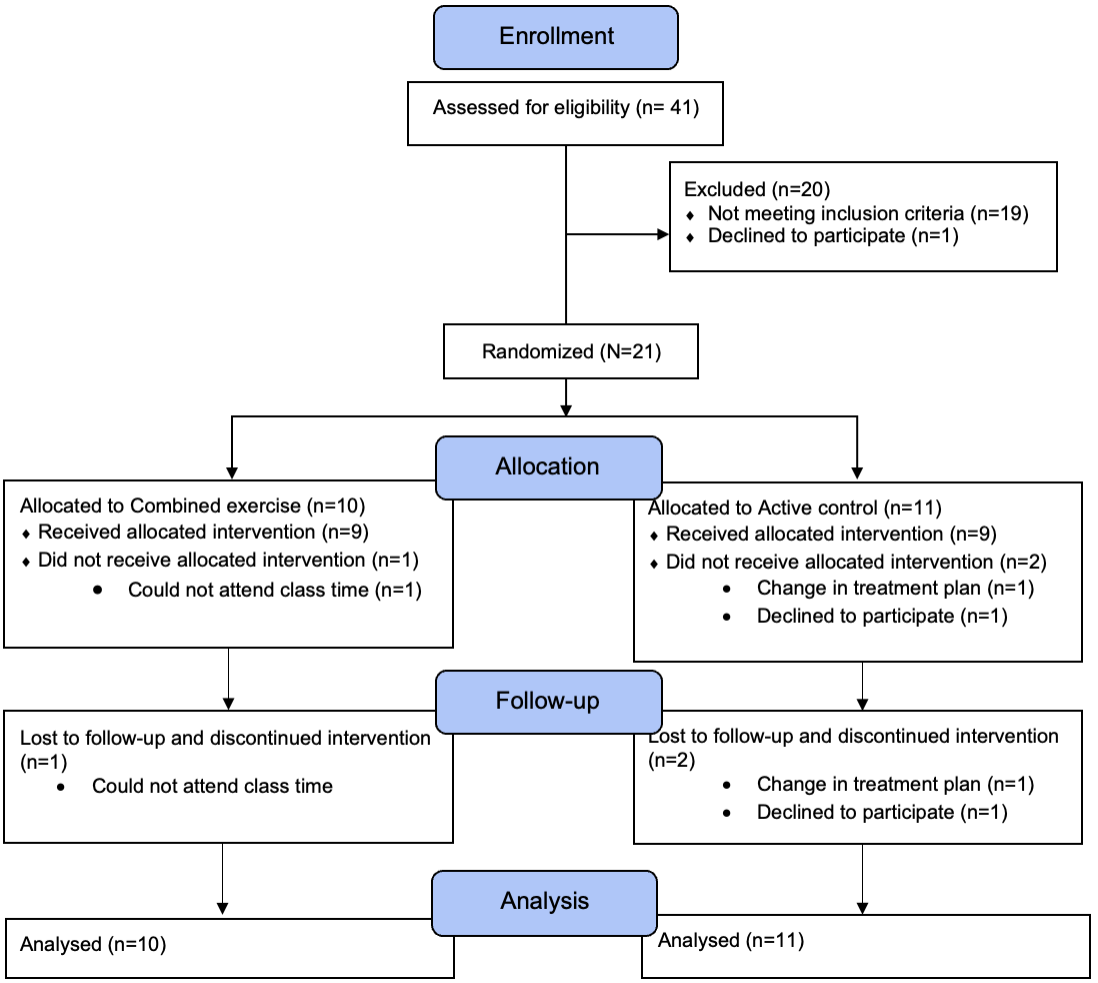
Participant flowchart through the study.

Participant flowchart is presented in Figure 2. Feasibility outcomes are summarized in Table 3. Of the 41 women who responded to the invitation, 22 were eligible, and 21 consented to be randomized into either the combined exercise group (aerobic+resistance training; n=10) or the active control group (balance and flexibility; n=11), representing a 51.2% enrollment rate. Participants were divided into two cohorts: (1) the first cohort (n=11) began in May 2023, and (2) the second cohort (n=10) began in July 2023. The sample size aligns with previous research on feasibility and group cohesion for counseling programs for patients with BC [70,71]. Overall, 18 participants completed the 8-week intervention. Three participants withdrew after randomization, but before starting the intervention for reasons, including a change in BC treatment plan (n=1), an issue with the timing of the programming and their schedule (n=1), and a change of interest in participating (n=1). An intention-to-treat analysis was conducted, and therefore adherence was calculated based on attendance and included the dropouts (n=3), since they were enrolled and randomized at the start of the intervention. Consequently, the attrition rate was 14.3%. For the ANCOVA analyses, a complete case analysis was applied (n=18).

**Table 3:** Feasibility and acceptability outcomes.

Feasibility variables	Definition	Adherence and completion, n/N (%)
Enrollment rate	Consented and randomized divided those invited	21/41 (51.2)
Attendance adherence (exercise sessions)	Attendance rate in exercise sessions	34/48 (70.8)
Attendance adherence (counseling sessions)^a^	Attendance rate in counseling sessions	31/40 (77.5)
Adverse events	Number of adverse events reported	0/0 (0)
Participant satisfaction questionnaire	Completion rate	18/21 (85.7)
Working Alliance Inventory	Completion rate	17/21 (81)
Remotely delivered NIH^b^ Toolbox Cognition Battery	Completion rate	18/21 (85.7)
PsyToolkit executive function test	Completion rate	17/21 (81)
Accelerometer wear compliance	Met wear-time criteria rate	14/21 (66.7)
Attrition	Dropouts after randomization	3/21 (14.3)

^a^Counseling sessions based on the multi-process action control were delivered only to participants assigned to the combined exercise group.

^a^NIH: National Institutes of Health.

Attendance adherence (Table 3) rate for the remotely delivered, combined exercise classes was 70.8% (17/24) and 77.5% (3/4) for behavioral counseling sessions based on the M-PAC that was delivered only to those in the combined exercise group (n=10). Attendance adherence for the active control group was 70.8% (17/24). Participants in the combined exercise group who completed the intervention and postintervention measures (n=9) reported an average RPE of 12.8 and a HR of 111.8 during the resistance training classes. This corresponds to a moderate-intensity RPE and an HR within the light-moderate target HR zone. In addition, participants in the combined exercise intervention (n=9) reported a mean RPE of 12.2 and a mean HR of 119.7 during the unsupervised aerobic (ie, walking) sessions, which corresponds to moderate-intensity RPE and a HR within the moderate-intensity target HR zone. Participants in the active control group who completed the intervention and postintervention measures (ie, including the exercise log; n=8) reported a mean RPE of 11.1 and a mean HR of 85.5 during their balance and flexibility program, which corresponds to a light-moderate intensity RPE and light-intensity target HR zone. There were no adverse events during the intervention in either group. The measurement completion rates for the study were as follows: 85.7% (18/21) for the participant satisfaction questionnaire and 81% (17/21) for the Working Alliance Inventory. For objectively measured cognitive function, completion rates were 85.7% (18/21) for the memory test and 81% (17/21) for the executive function test. Additionally, accelerometer wear compliance was achieved by 66.7% (14/21) of participants.

Participant responses regarding program satisfaction are summarized in Tables 4 and 5. Overall, participants reported high satisfaction with the intervention. The majority of participants (17/18, 94.4%) agreed or strongly agreed that the program was rewarding and appreciated the virtual delivery format. Most participants (15/18, 83.3%) agreed or strongly agreed that the intervention helped them increase their PA levels, and 77.7% (14/18) indicated that they would recommend the program to other patients with BC. Most participants found the intervention beneficial, and participants 62.5%) agreed or strongly agreed that the behavioral counseling sessions were personally useful. Similarly, 61.1% of participants agreed or strongly agreed that the intervention was not burdensome.

**Table 4:** Satisfaction scores from the participant satisfaction questionnaire in patients with breast cancer at post intervention (ie, 8 weeks; n=18).

Variable		Value, mean (SD)	Strongly agree and agree (%)
**Overall intervention participation**			
	I found the exercise intervention rewarding.	4.6 (0.6)	94.4
	I enjoyed the virtual delivery of the exercise intervention.	4.6 (0.6)	94.4
	The exercise intervention helped me to increase my physical activity levels.	4.0 (1.1)	83.3
	The exercise intervention helped me to increase my overall fitness.	3.9 (1.1)	72.2
	The behavioral counseling sessions were useful for me personally.	3.1 (1.5)	62.5
	I am more confident in my goal-setting and coping abilities after participating in the intervention.	3.7 (1.1)	61.1
	The research study was useful for me personally.	3.7 (1.0)	83.3
	I would recommend the exercise intervention to other patients with breast cancer.	4.2 (0.7)	77.7

**Table 5:** Burden scores from the participant satisfaction questionnaire in patients with breast cancer at post intervention (ie, 8 weeks; n=18).

Burden	Value, mean (SD)	Strongly disagree and disagree (%)
The exercise intervention was a waste of time.	1.8 (1.3)	83.3
The behavioral counseling sessions were a waste of time.	2.3 (1.5)	75.0
The questionnaires were a burden.	2.3 (1.2)	61.1
The neurological assessments (iPad cognition tests) were a burden.	2.1 (1.1)	77.8
The 3-times weekly exercise class was a burden.	2.1 (0.9)	77.8
It was difficult to participate in the exercise classes because of cancer or chemotherapy-related side effects.	2.5 (1.3)	55.6

Participant responses on the WAI-SR are summarized in Table 6. Overall, participants reported positive perceptions of their alliance with the QEP. Most (16/18, 88.2%) indicated that they very often or always felt mutual respect, and a substantial majority (14/18, 76.5%) felt appreciated and cared for.

**Table 6:** Working alliance inventory-short revised scores in patients with breast cancer post intervention (ie, 8 weeks; n=17)^a^.

Variable	Value, mean (SD)	Very often or always (%)
As a result of these sessions, I am clearer as to how I might be able to change.	2.9 (1.2)	35.3
What I am doing in therapy gives me new ways of looking at my problem.	3.2 (1.3)	41.2
I believe [INSTRUCTOR] likes me.	4.1 (0.8)	70.6
[INSTRUCTOR] and I collaborate on setting goals for my therapy.	2.9 (1.4)	35.3
[INSTRUCTOR] and I respect each other.	4.5 (0.7)	88.2
[INSTRUCTOR] and I are working towards mutually agreed upon goals.	3.2 (1.2)	47.1
I feel that [INSTRUCTOR] appreciates me.	4.2 (0.9)	76.5
[INSTRUCTOR] and I agree on what is important for me to work on.	3.2 (1.2)	35.3
I believe the way we are working with my problem is correct.	3.7 (1.3)	64.7
I feel that the things I do in therapy will help me to accomplish the changes that I want.	3.6 (1.3)	64.7
[INSTRUCTOR] and I have established a good understanding of the kind of changes that would be good for me.	3.1 (1.5)	41.2
I feel [INSTRUCTOR] cares about me even when I do things that he/she does not approve of.	4.2 (0.9)	76.5

^a^Participants rated items on a 5-point Likert scale, where 1=seldom, 2=sometimes, 3=fairly often, 4=very often, and 5=always. Participants were told to replace the term “therapy” with “exercise program” when thinking about their responses to each item.

### Objectively Measured Cognitive Outcomes

Changes in objectively measured cognitive function scores from baseline to post intervention for the combined exercise group vs the active control group are presented in Table 7. There were no statistical differences in objectively measured cognitive function in the combined exercise group compared to the active control group. However, adjusted group mean differences trending toward the combined exercise group were observed in the scores for the Picture Sequence Memory Task (mean difference +5.33, 95% CI –12.5 to 23.2; ηp²=0.03), List Sorting Working Memory Test (mean difference +8.17, 95% CI –4.2 to 20.6; ηp²=0.12), and Auditory Verbal Learning (mean difference +3.22, 95% CI –2.0 to 8.5; ηp²=0.12). Table 7 also shows the 4 cognition outcomes from the PsyToolkit: repeat trial time (ms), switch trial time (ms), repeat trials correct (%), and switch trials correct (%). No significant differences were found between the combined exercise group compared to the active control group. However, a reduction in repeat trial time trended toward the combined exercise group (mean difference –146.14, 95% CI 420.87 to128.59 ms; ηp²=0.04). Likewise, a greater decrease in the switch trial time trended toward the combined exercise group (mean difference –394.35, 95% CI –1035.67 to 246.96 ms; ηp²=0.07) compared to the control group (mean difference –124.39 95% CI –522.06 to 273.27 ms; ηp²=0.07). For accuracy, improvements in repeat trials correct (mean difference +4.86%, 95% CI –0.61 to 10.36; ηp²=0.02) and switch trials correct (mean difference +19.70%, –0.30 to 39.70; ηp²=0.02) also trended towards the combined exercise group, while the control group exhibited decreases in these measures.

**Table 7:** Effects of a supervised, remotely delivered, combined exercise or active control group on cognitive function at post intervention (n=18).

Variable			Pre, mean (SD)		Post, mean (SD)		Mean difference (95% CI)			*P* value			Partial η²	
**Picture sequence memory**								5.33 (–12.5 to 23.2)			.53			0.03
	Combined exercise (n=9)	107.1 (12.1)		112.2 (16.6)										
	Active control (n=9)	108.4 (18.8)		107.6 (22.0)										
**List sorting memory**								8.17 (–4.2 to 20.6)			.18			0.12
	Combined exercise (n=9)	106.4 (16.1)		111.4 (13.3)										
	Active control (n=9)	100.7 (17.7)		102.0 (11.2)										
**Auditory verbal learning test**								3.22 (–2.0 to 8.5)			.21			0.12
	Combined exercise (n=9)	28.5 (5.9)		32.0 (6.7)										
	Active control (n=9)	26.1 (7.0 )		26.6 (8.2)										
**Oral reading recognition**								–9.65 (–22.9 to 3.5)			.14			0.14
	Combined exercise (n=9)	129.8 (22.4)		127.7 (20.4)										
	Active control (n=9)	128.0 (15.01)		136.4 (9.0)										
**Picture vocabulary**								–2.48 (–5.2 to 0.3)			.07			0.20
	Combined exercise (n=9)	103.7 (14.5)		102.0 (15.0)										
	Active control (n=9)	106.0 (12.0)		106.7 (11.6)										
**Repeat trials reaction time (ms)**										.42			0.04	
	Combined exercise (n=9)	1266.23 (86.29)		1120.09 (88.92)		–146.14 (–420.87 to128.59)								
	Active control (n=8)	1119.22 (68.91)		1179.75 (91.80)		60.53 (–132.71 to 253.78)								
**Switch trials reaction time (ms)**										.34			0.07	
	Combined exercise (n=9)	2268.51 (205.67)		1874.16 (123.64)		–394.35 (–1035.67 to 246.96)								
	Active control (n=8)	2227.06 (114.37)		2102.67 (187.89)		–124.39 (–522.06 to273.27)								
**Percentage of correct repeat trials (%)**											.62			0.02
	Combined exercise (n=9)	90.59 (1.61)		95.46 (1.67)		4.86 (–0.61 to 10.36)								
	Active control (n=8)	96.68 (0.92)		92.86 (2.66)		–3.83 (–10.62 to 2.96)								
**Percentage of correct switch trials (%)**											.63			0.02
	Combined exercise (n=9)	69.70 (6.86)		89.39 (2.83)		19.70 (–0.30 to39.70)								
	Active control (n=8)	92.05 (2.06)		81.25 (5.26)		–10.80 (–24.26 to 2.67)								

Table 8 presents the changes in device-based exercise levels among patients with BC. There were no statistical differences between the two groups. However, an increase in total moderate-to-vigorous intensity exercise minutes was observed from pre to post intervention, trending toward participants in the active control group compared to those in the combined exercise group (mean difference in change 58.8, 95% CI 105.8.8-223.6 minutes; ηp²=0.06).

**Table 8:** Effects of supervised, remotely delivered, combined exercise or active control program on total moderate-to-vigorous exercise in patients with breast cancer at pre and post intervention (ie, 8 weeks; n=14).

Variable			Pre intervention, mean (SD)		Post intervention, mean (SD)		Adjusted mean difference between groups^a^							
							Mean difference (95% CI)			*P* value			Partial *n*^2^	
**Total moderate-to-vigorous intensity exercise**								58.8 (105.8.8-223.6)			.44			0.06
	Active control (n=6)	179.3 (110.3)		272.0 (230.6)										
	Combined exercise (n=8)	127.0 (96.0)		148.5 (162.9)										
**Light-intensity exercise**								74.5 (–625.0 to 774.1)			.82			0.15
	Active control (n=6)	1940.3 (431.9)		1991.6 (319.9)										
	Combined exercise (n=8)	2140.4 (388.8)		2168.3 (555.5)										
**Moderate-intensity exercise**								26.6 (–89.8 to 143.0)			.62			0.02
	Active control (n=6)	158.6 (89.8)		211.6 (153.3)										
	Combined exercise (n=8)	123.2 (88.1)		144.1 (154.5)										
**Vigorous-intensity exercise**								48.2 (–6.6 to 103.0)			.08			0.28
	Active control (n=6)	17.3 (41.9)		55.2 (71.1)										
	Combined exercise (n=8)	3.75 (10.6)		4.25 (9.01)										

^a^Mean differences were adjusted for the baseline value of the outcome and total minutes of wear time.

## Discussion

### Principal Findings

To our knowledge, this is the first study to pilot a remotely delivered, supervised combined exercise program for patients with BC following chemotherapy, exhibiting mild cognitive impairment. The findings of this study will be used to inform larger, definitive RCTs by examining which aspects of the trial were feasible, as well as further refinements that need to be made. Specifically, there are few exercise training studies that target BC treatment-related cognitive impairment as a primary outcome. Recent reviews examined evidence for the use of exercise as an intervention for CRCI [25,31,72]. However, these studies did not consistently measure cognitive function as a primary outcome, and less than half assessed cognitive function with well-normed objective measures. The varied results within neuropsychological testing and the recent emphasis on individualized care all highlight the critical need to identify patients with BC who are especially at risk for cognitive decline. Our pilot is noteworthy, as it addressed some of these conceptual and methodological issues that may advance progress in the field. These include having an active control condition to match contact time, examining different types of exercise, such as combined exercise, including computer-based objectively measured cognitive function, as well as theory-based, behavioral counseling for exercise uptake and adherence.

This study was feasible and exceeded the a priori feasibility indicators of adherence rate ≥70%, attrition rate <30%, and no adverse events, and was well received by patients with BC. Adherence rates in our remotely delivered trial were consistent (63%-100% [28,30,73]) or lower (88% [72]; 87.9% [26]; 80% [74]; 99.9% [75]) than previous exercise trials targeting CRCI. However, it is important to note that these exercise trials were supervised (in person) sessions that included different exercise modalities, such as high-intensity interval training [28,73], combined exercise with Nordic or power walking [30], home-based walking [71], and aerobic exercise [26,72,75]. Nevertheless, our adherence rates are encouraging when compared to other virtually supervised exercise interventions delivered via Zoom in a group format, with various combinations of aerobic and resistance exercises, where adherence ranged from 78% to 100% [76]. Our trial also had low attrition rates, which were consistent with prior research with remotely delivered exercise interventions (0%-29%; [76]).

Recruitment rates for virtually supervised exercise interventions vary widely, with reported rates between 8% and 60% [76] likely due to the heterogeneity of recruitment methods. While our recruitment rate (51.2%) was consistent with previous research, our study relied mainly on self-referrals, which tended to attract highly motivated cancer survivors who were well-educated, White, and in better overall health and physical functioning. Homogeneous samples (eg, White, young, and highly educated) are a common limitation across distance-based exercise oncology trials [77]. Recruitment strategies and partnerships with organizations that serve racial and ethnic populations of cancer survivors should be strengthened at the onset of the intervention, which may assist with recruitment efforts [78].

In our secondary outcomes, no statistical differences were observed in objectively measured cognitive function. Small to medium effect size improvements were observed in episodic memory (Picture Sequence Memory Task), working memory, and executive function updating (List Sorting Working Memory Test), and immediate memory and verbal learning (Auditory Verbal Learning), trending toward the combined exercise group. On the other hand, large effect size improvements were observed in crystallized abilities (Oral Reading Recognition Test) plus language and vocabulary (Picture Vocabulary), slightly trending toward the active control group. For executive function shifting, greater accuracy and reductions in reaction time (small to medium effect sizes) were observed for the switch trials in the combined exercise group. To date, few studies have tested the effects of exercise on objectively measured cognitive function outcomes in patients with BC following chemotherapy, with mixed results [40,72,75]. Preliminary results from these trials support cognitive changes following an exercise intervention, which is consistent with a study that examined the effects of a 3-month PA intervention compared to a waitlist control arm on cognitive functioning in patients with BC [43]. The intervention improved processing speed only among patients with BC diagnosed within the past 2 years. One proof-of-concept aerobic exercise intervention examined cognitive function using objective neuropsychological testing and functional magnetic resonance imaging in patients with BC. This study found improvements in cognitive symptoms with notable patterns of neural activation. However, the changes either did not reach statistical significance or had small effect sizes [72]. Finally, one of the first large-scale studies to examine the effects of aerobic exercise on neurocognitive function tested the effects of 6 months of moderate-intensity aerobic exercise on neurocognitive function in patients with BC receiving endocrine therapy. The trial focused on women who initiate exercise within 2 years of completing primary therapy (surgery +/− chemotherapy) [75]. Findings show improved performance in processing speed and verbal memory in the exercise group and no change in the usual care control group. It is important to note that these studies were either home-based aerobic programs or in person supervised aerobic activity. They also used a wide range of cognitive measures that were collected in person, whereas our study used a cognitive battery that was assessed remotely.

Recent reviews on the use of exercise as a potential intervention for cognitive deficits associated with cancer and its treatment demonstrated notable improvements in the cognitive domains of working and visual memory, attention and concentration, cognitive flexibility, inhibitory control, and verbal fluency [26,79], consistent with our study. Null effects were observed in other RCTs with a walking intervention [74], exercise intervention with supervised aerobic and strength training, as well as Nordic or power walking [30], and interval and continuous aerobic exercise in patients with BC following chemotherapy [73]. While findings from previous trials have been inconclusive, the positive improvements in cognitive function reported in our study could be attributed to recruiting patients with BC who exhibit mild cognitive impairment at the onset of the intervention. This may have provided greater room for improvement post intervention. Also, a range of cognitive assessments measuring cognitive domains that are most impaired by chemotherapy (eg, memory, attention, and executive function) increased the sensitivity to detect impairments post intervention. Finally, patients with BC in the combined exercise intervention were adhering to the prescribed exercise dose (ie, moderate-to-vigorous intensity physical activity [MVPA]), which is critical in achieving greater cognitive benefits and is consistent with reviews demonstrating that sustained MVPA elicits changes in cognitive function in patients with BC [3,25,80]. One of the most important mediators of cognition in patients with BC may be cardiorespiratory fitness [81,82]. Exercise-induced changes in cognitive function can be explained by alterations in brain morphology and functional dynamics (ie, prefrontal cortex and hippocampus) and may be partially mediated by improvements in cardiorespiratory fitness [82]. This underscores the pivotal role of cardiorespiratory fitness in amplifying the neuroprotective effects of MVPA. Given the link between cognition and cardiorespiratory fitness and the established effects of exercise on cardiorespiratory fitness, exercise might also remediate CRCI in patients with BC.

Notable improvements were also observed in the active control group performing light-intensity exercise, but these improvements were not in the cognitive domains most impaired by chemotherapy. The active control group engaged in a light-intensity exercise, which is insufficient to elicit improvements in cardiorespiratory fitness. Previous reviews demonstrated that both aerobic and resistance training that were moderate-to-vigorous intensity may be required to elicit cognitive benefits [3]. Nevertheless, improvements in the Oral Reading Recognition Test and Picture Vocabulary Test trended toward the active control group (although exploratory). These domains are related to language and/or crystallized abilities, and it is possible that increases in cognitive performance likely reflect expectancy or practice effects from repeated exposure to the assessments. Therefore, the remote administration of the NIH Toolbox Cognition Battery may not fully capture subtle CRCI-related changes.

In terms of changes in exercise minutes, improvements in MVPA from pre to post intervention trended toward the active control group (although exploratory). This finding is inconsistent with previous studies, which demonstrated improvements in device-based exercise minutes in patients with BC [30,43]. Given that we had an active control group, both groups were motivated to engage in exercise. Accelerometers do not accurately measure resistance training, which may not have been captured in patients with BC, especially in the combined exercise group, who continued engaging in resistance training. Patients with BC in the combined exercise group may have engaged in less structured exercise outside of the planned classes because they were receiving a moderate-intensity multicomponent exercise program compared to the active control group. Therefore, the reported effect sizes between our groups are possibly smaller given the active comparator group. Also, patients with BC self-reported not meeting exercise guidelines at screening but subsequently showed higher levels of activity with device-based measures at baseline. Future research should consider using device-based measures to screen patients with BC not meeting exercise guidelines to ensure that the inclusion criteria are met.

### Lessons Learned and Future Research Directions

Overall, patients with BC were highly satisfied with the intervention. Most patients with BC found the study rewarding, enjoyed the virtual delivery format, would recommend the study to other patients with BC, and did not find the study assessments burdensome. Patients with BC also felt a mutual respect with their QEP and felt appreciated by their QEP, further contributing to their positive experience. This suggests that few adjustments need to be made to the intervention or the study design before implementing a future RCT. In our study, an additional QEP attended the classes to monitor participant safety and to assist the lead QEP in managing the virtual environment. This was beneficial to both the lead QEP and patients with BC, and future remotely delivered interventions should consider a second QEP. Additionally, the QEP’s personality, approachability, and teaching style played an important role in the enjoyment and feasibility for patients with BC. Future studies should consider QEPs with cancer-specific training to offer exercise modifications to patients with BC with limitations or injuries. Finally, our classes included 15 minutes of optional time before or after each class for patients with BC to interact for overall class enjoyment and accountability and to help foster a sense of community. This was beneficial to the overall satisfaction of the trial and should be implemented in future studies.

### Strengths and Limitations

Our trial should be interpreted within the context of important strengths and limitations. The remotely delivered component of the trial was a strength, as it removed preexisting barriers to exercise (eg, time constraints, access, and hesitancy toward public space). The combined exercise and active control group sessions were delivered by knowledgeable QEPs who were engaging and provided modifications that allowed all patients with BC to participate in exercise, despite any physical limitations. The addition of a recorded session reduced participant burden by providing flexibility and autonomy. Our study was strengthened by the inclusion of behavioral counseling sessions, which were guided by the M-PAC framework to encourage exercise adoption and adherence [51]. The inclusion of a rigorous comparison group was an additional strength since both groups received supervised, remotely delivered exercise programs at differing intensities. Finally, the use of a standardized, well-validated battery of cognitive tests via the NIH Toolbox was another strength and in line with the ICCTF’s recommendations [6].

Although this study had a small sample size and a heterogeneous population, which limits the generalizability of the findings, this feasibility study is crucial for calculating sample sizes and refining methodologies for larger, future trials. In addition, the sample was predominantly White, highly educated, middle- to older-aged, and within the early cancer survivorship period. The focus on early-stage patients with BC within 48 months of completing treatment may not be generalizable to other cancer populations, such as individuals with metastatic disease or patients who are pretreatment, undergoing treatment, or immediately post treatment. Furthermore, the heterogeneity in the time since treatment in our study may preclude identifying the optimal exercise timing to prevent or ameliorate CRCI, which requires future investigation. It is important to note that patients with BC may be more likely to seek out care for cancer rehabilitation and view themselves as research participants compared to patients with BC who are more culturally and socially diverse populations. Future research should consider the recommendations for increasing racial and ethnic diversity in cancer clinical trials by the American Society of Clinical Oncology and Association of Community Cancer Centers Joint Research Statement [78]. Another limitation of this study was not accounting for menopausal status, which could be a confounding variable that may impact cognitive function in patients with BC. Future studies should consider chemotherapy-induced menopause as a possible mechanism for CRCI. Furthermore, our study did not include self-reported cognitive function, which is another important indicator of CRCI, as it taps into day-to-day cognitive performance in relation to cancer and its treatments [6,83]. Therefore, including both self-report and objective cognitive function assessments in future research is warranted.

### Conclusions

In conclusion, this feasibility study provides preliminary evidence that a remotely delivered, combined exercise program is feasible and may improve objectively measured cognitive function. There are a few exercise studies that have rigorously evaluated CRCI as a primary outcome in patients with BC. The limited number of studies conducted makes it difficult to draw conclusions about the efficacy of exercise for improving cognitive function in patients with BC. There is a need for rigorous and well-designed RCTs. While the use of shorter exercise protocols like our study provides preliminary evidence for cognitive benefits, long-term exercise is more efficacious in addressing age-related cognitive decline and improving cognition [84]. Future trials should include active control conditions, test multicomponent exercises (ie, aerobic and resistance training), and examine the mechanisms underlying the relationship between exercise and cognitive function. Given that CRCI is an emerging cancer survivorship issue, the next step for this work is to conduct a larger RCT that is adequately powered to examine the effects of a remotely delivered, combined exercise intervention on cognitive function in patients with BC following chemotherapy. If replicated, these findings have the potential to identify effective and modifiable management strategies to enhance cognitive health in patients with BC.

## Appendix

Multimedia Appendix 1 CONSORT-eHEALTH checklist (V 1.6.1).

## Abbreviations

ACSM: American College of Sports Medicine
ACTIVATE: Aerobic exercise and CogniTIVe functioning in women with breAsT cancEr
ANCOVA: analyses of covariance
BC: breast cancer
CRCI: cancer-related cognitive impairment
CSEP: Canadian Society of Exercise Physiologists
CONSORT-EHEALTH: Consolidated Standards of Reporting Trials of Electronic and Mobile Health Applications and Online Telehealth
HR: heart rate
ICCTF: International Cognition and Cancer Task Force
MVPA: moderate-to-vigorous intensity physical activity
M-PAC: multi-process action control framework
maSCD: menopause‐associated subjective cognitive decline
NIH: National Institutes of Health
PA: physical activity
QEP: Qualified Exercise Professional
QoL: quality of life
RCT: randomized controlled trial
REDCap: Research Electronic Data Capture
RPE: rating of perceived exertion
TICS-M: Modified Telephone Interview of Cognitive Status
WAI-SR: Working Alliance Inventory – Short Revised

## Data Availability

The datasets generated and analyzed during this study are not publicly available due to the sensitive information of patients but are available from the corresponding author on reasonable request.
