# Supplementary material for: The Feasibility and Acceptability of a Remotely Delivered, Combined Exercise Intervention on Cognitive Function in Patients With Breast Cancer Following Chemotherapy: Randomized Controlled Trial
Source: JMIR Cancer. 2026 Feb 23;12:e73393. doi: 10.2196/73393 (PMC12972685; doi:10.2196/73393)
Supplement: Multimedia Appendix 1 [file cancer_v12i1e73393_app1.pdf]

# CONSORT-EHEALTH (V 1.6.1) - Submission/Publication Form

The CONSORT-EHEALTH checklist is intended for authors of randomized trials evaluating web-based and Internet-based applications/interventions, including mobile interventions, electronic games (incl multiplayer games), social media, certain telehealth applications, and other interactive and/or networked electronic applications. Some of the items (e.g. all subitems under item 5 - description of the intervention) may also be applicable for other study designs.

The goal of the CONSORT EHEALTH checklist and guideline is to be
a) a guide for reporting for authors of RCTs,
b) to form a basis for appraisal of an ehealth trial (in terms of validity)

CONSORT-EHEALTH items/subitems are MANDATORY reporting items for studies published in the Journal of Medical Internet Research and other journals / scientific societies endorsing the checklist.

Items numbered 1., 2., 3., 4a., 4b etc are original CONSORT or CONSORT-NPT (non-pharmacologic treatment) items.
Items with Roman numerals (i., ii, iii, iv etc.) are CONSORT-EHEALTH extensions/clarifications.

As the CONSORT-EHEALTH checklist is still considered in a formative stage, we would ask that you also RATE ON A SCALE OF 1-5 how important/useful you feel each item is FOR THE PURPOSE OF THE CHECKLIST and reporting guideline (optional).

Mandatory reporting items are marked with a red *.
In the textboxes, either copy & paste the relevant sections from your manuscript into this form - please include any quotes from your manuscript in QUOTATION MARKS,
or answer directly by providing additional information not in the manuscript, or elaborating on why the item was not relevant for this study.

YOUR ANSWERS WILL BE PUBLISHED AS A SUPPLEMENTARY FILE TO YOUR PUBLICATION IN JMIR AND ARE CONSIDERED PART OF YOUR PUBLICATION (IF ACCEPTED).
Please fill in these questions diligently. Information will not be copyedited, so please use proper spelling and grammar, use correct capitalization, and avoid abbreviations.

DO NOT FORGET TO SAVE AS PDF _AND_ CLICK THE SUBMIT BUTTON SO YOUR ANSWERS ARE IN OUR DATABASE !!!

Citation Suggestion (if you append the pdf as Appendix we suggest to cite this paper in the caption):
Eysenbach G, CONSORT-EHEALTH Group

CONSORT-EHEALTH: Improving and Standardizing Evaluation Reports of Web-based and Mobile Health Interventions
J Med Internet Res 2011;13(4):e126
URL: http://www.jmir.org/2011/4/e126/
doi: 10.2196/jmir.1923
PMID: 22209829

Sign in to Google to save your progress. Learn more

* Indicates required question

Your name *

First Last

Linda Trinh

Primary Affiliation (short), City, Country *

University of Toronto, Toronto, Canada

University of Toronto, Toronto, Canada

Your e-mail address *

abc@gmail.com

linda.trinh@utoronto.ca

Title of your manuscript *

Provide the (draft) title of your manuscript.

The Feasibility and Acceptability of a Remotely-Delivered, Combined Exercise Intervention on Cognitive Function in Breast Cancer Patients Following Chemotherapy: A Randomized Controlled Trial

Name of your App/Software/Intervention *

If there is a short and a long/alternate name, write the short name first and add the long name in brackets.

not applicable

Evaluated Version (if any)

e.g. "V1", "Release 2017-03-01", "Version 2.0.27913"

not applicable

Language(s) *

What language is the intervention/app in? If multiple languages are available, separate by comma (e.g. "English, French")

English

URL of your Intervention Website or App

e.g. a direct link to the mobile app on app in appstore (itunes, Google Play), or URL of the website. If the intervention is a DVD or hardware, you can also link to an Amazon page.

https://kpe.utoronto.ca/academics-researchresearch-units-labs-centres/exercise-oncology-la

URL of an image/screenshot (optional)

Your answer

Accessibility *

Can an enduser access the intervention presently?

- ( ) access is free and open
- ( ) access only for special usergroups, not open
- ( ) access is open to everyone, but requires payment/subscription/in-app purchases
- (x) app/intervention no longer accessible
- ( ) Other:

Primary Medical Indication/Disease/Condition *

e.g. "Stress", "Diabetes", or define the target group in brackets after the condition, e.g. "Autism (Parents of children with)", "Alzheimers (Informal Caregivers of)"

breast cancer

Primary Outcomes measured in trial *

comma-separated list of primary outcomes reported in the trial

feasibility (enrollment rate, attrition rates, adhe

Secondary/other outcomes

Are there any other outcomes the intervention is expected to affect?

objective  measures of attention, executive function, and memory

Recommended "Dose" *

What do the instructions for users say on how often the app should be used?

- ( ) Approximately Daily
- (x) Approximately Weekly
- ( ) Approximately Monthly
- ( ) Approximately Yearly
- ( ) "as needed"
- ( ) Other:

Approx. Percentage of Users (starters) still using the app as recommended after 3 months *

- (x) unknown / not evaluated
- ( ) 0-10%
- ( ) 11-20%
- ( ) 21-30%
- ( ) 31-40%
- ( ) 41-50%
- ( ) 51-60%
- ( ) 61-70%
- ( ) 71%-80%
- ( ) 81-90%
- ( ) 91-100%
- ( ) Other:

Overall, was the app/intervention effective? *

- ○ yes: all primary outcomes were significantly better in intervention group vs control
- ◉ partly: SOME primary outcomes were significantly better in intervention group vs control
- ○ no statistically significant difference between control and intervention
- ○ potentially harmful: control was significantly better than intervention in one or more outcomes
- ○ inconclusive: more research is needed
- ○ Other:

Article Preparation Status/Stage *

At which stage in your article preparation are you currently (at the time you fill in this form)

- ○ not submitted yet - in early draft status
- ○ not submitted yet - in late draft status, just before submission
- ○ submitted to a journal but not reviewed yet
- ◉ submitted to a journal and after receiving initial reviewer comments
- ○ submitted to a journal and accepted, but not published yet
- ○ published
- ○ Other:

Journal *

If you already know where you will submit this paper (or if it is already submitted), please provide the journal name (if it is not JMIR, provide the journal name under "other")

- ◯ not submitted yet / unclear where I will submit this
- ◯ Journal of Medical Internet Research (JMIR)
- ◯ JMIR mHealth and UHealth
- ◯ JMIR Serious Games
- ◯ JMIR Mental Health
- ◯ JMIR Public Health
- ◯ JMIR Formative Research
- ◉ Other JMIR sister journal
- ◯ Other:

Is this a full powered effectiveness trial or a pilot/feasibility trial? *

- ◉ Pilot/feasibility
- ◯ Fully powered

Manuscript tracking number *

If this is a JMIR submission, please provide the manuscript tracking number under "other" (The ms tracking number can be found in the submission acknowledgement email, or when you login as author in JMIR. If the paper is already published in JMIR, then the ms tracking number is the four-digit number at the end of the DOI, to be found at the bottom of each published article in JMIR)

- ◯ no ms number (yet) / not (yet) submitted to / published in JMIR
- ◉ Other: 73393

## TITLE AND ABSTRACT

### 1a) TITLE: Identification as a randomized trial in the title

### 1a) Does your paper address CONSORT item 1a? *

I.e does the title contain the phrase "Randomized Controlled Trial"? (if not, explain the reason under "other")

- (•) yes
- ( ) Other:

### 1a-i) Identify the mode of delivery in the title

Identify the mode of delivery. Preferably use “web-based” and/or “mobile” and/or “electronic game” in the title. Avoid ambiguous terms like “online”, “virtual”, “interactive”. Use “Internet-based” only if Intervention includes non-web-based Internet components (e.g. email), use “computer-based” or “electronic” only if offline products are used. Use “virtual” only in the context of “virtual reality” (3-D worlds). Use “online” only in the context of “online support groups”. Complement or substitute product names with broader terms for the class of products (such as “mobile” or “smart phone” instead of “iphone”), especially if the application runs on different platforms.

| | 1 | 2 | 3 | 4 | 5 | |
|---|---|---|---|---|---|---|
| subitem not at all important | ( ) | ( ) | ( ) | ( ) | (•) | essential |

Clear selection

Does your paper address subitem 1a-i? *

Copy and paste relevant sections from manuscript title (include quotes in quotation marks "like this" to indicate direct quotes from your manuscript), or elaborate on this item by providing additional information not in the ms, or briefly explain why the item is not applicable/relevant for your study

The Feasibility and Acceptability of a Remotely-Delivered, Combined Exercise Intervention on Cognitive Function in Breast Cancer Patients Following Chemotherapy:
A Randomized Controlled Trial

1a-ii) Non-web-based components or important co-interventions in title

Mention non-web-based components or important co-interventions in title, if any (e.g., “with telephone support”).

| | 1 | 2 | 3 | 4 | 5 | |
|---|---|---|---|---|---|---|
| subitem not at all important | ○ | ○ | ○ | ◉ | ○ | essential |

Clear selection

Does your paper address subitem 1a-ii?

Copy and paste relevant sections from manuscript title (include quotes in quotation marks "like this" to indicate direct quotes from your manuscript), or elaborate on this item by providing additional information not in the ms, or briefly explain why the item is not applicable/relevant for your study

The Feasibility and Acceptability of a Remotely-Delivered, Combined Exercise Intervention on Cognitive Function in Breast Cancer Patients Following Chemotherapy:
A Randomized Controlled Trial

### 1a-iii) Primary condition or target group in the title

Mention primary condition or target group in the title, if any (e.g., “for children with Type I Diabetes”) Example: A Web-based and Mobile Intervention with Telephone Support for Children with Type I Diabetes: Randomized Controlled Trial

| | 1 | 2 | 3 | 4 | 5 | |
|---|---|---|---|---|---|---|
| subitem not at all important | ○ | ○ | ○ | ○ | ◉ | essential |

Clear selection

### Does your paper address subitem 1a-iii? *

Copy and paste relevant sections from manuscript title (include quotes in quotation marks "like this" to indicate direct quotes from your manuscript), or elaborate on this item by providing additional information not in the ms, or briefly explain why the item is not applicable/relevant for your study

The Feasibility and Acceptability of a Remotely-Delivered, Combined Exercise Intervention on Cognitive Function in Breast Cancer Patients Following Chemotherapy:
A Randomized Controlled Trial

### 1b) ABSTRACT: Structured summary of trial design, methods, results, and conclusions

NPT extension: Description of experimental treatment, comparator, care providers, centers, and blinding status.

1b-i) Key features/functionalities/components of the intervention and comparator in the METHODS section of the ABSTRACT

Mention key features/functionalities/components of the intervention and comparator in the abstract. If possible, also mention theories and principles used for designing the site. Keep in mind the needs of systematic reviewers and indexers by including important synonyms. (Note: Only report in the abstract what the main paper is reporting. If this information is missing from the main body of text, consider adding it)

| | 1 | 2 | 3 | 4 | 5 | |
|---|---|---|---|---|---|---|
| subitem not at all important | ○ | ○ | ○ | ○ | ◉ | essential |

Clear selection

Does your paper address subitem 1b-i? *

Copy and paste relevant sections from the manuscript abstract (include quotes in quotation marks "like this" to indicate direct quotes from your manuscript), or elaborate on this item by providing additional information not in the ms, or briefly explain why the item is not applicable/relevant for your study

This study examined the feasibility of delivering an 8-week remotely-delivered combined exercise program (aerobic + resistance training) compared to a stretching/toning active control in BC patients following chemotherapy.

1b-ii) Level of human involvement in the METHODS section of the ABSTRACT

Clarify the level of human involvement in the abstract, e.g., use phrases like “fully automated” vs. “therapist/nurse/care provider/physician-assisted” (mention number and expertise of providers involved, if any). (Note: Only report in the abstract what the main paper is reporting. If this information is missing from the main body of text, consider adding it)

| | 1 | 2 | 3 | 4 | 5 | |
|---|---|---|---|---|---|---|
| subitem not at all important | ○ | ○ | ○ | ◉ | ○ | essential |

Clear selection

Does your paper address subitem 1b-ii?

Copy and paste relevant sections from the manuscript abstract (include quotes in quotation marks "like this" to indicate direct quotes from your manuscript), or elaborate on this item by providing additional information not in the ms, or briefly explain why the item is not applicable/relevant for your study

BC patients who completed (neo)adjuvant chemotherapy within 48 months were recruited across Canada from February to July 2023.

1b-iii) Open vs. closed, web-based (self-assessment) vs. face-to-face assessments in the METHODS section of the ABSTRACT

Mention how participants were recruited (online vs. offline), e.g., from an open access website or from a clinic or a closed online user group (closed usergroup trial), and clarify if this was a purely web-based trial, or there were face-to-face components (as part of the intervention or for assessment). Clearly say if outcomes were self-assessed through questionnaires (as common in web-based trials). Note: In traditional offline trials, an open trial (open-label trial) is a type of clinical trial in which both the researchers and participants know which treatment is being administered. To avoid confusion, use “blinded” or “unblinded” to indicated the level of blinding instead of “open”, as “open” in web-based trials usually refers to “open access” (i.e. participants can self-enrol). (Note: Only report in the abstract what the main paper is reporting. If this information is missing from the main body of text, consider adding it)

| | 1 | 2 | 3 | 4 | 5 | |
|---|---|---|---|---|---|---|
| subitem not at all important | ○ | ○ | ○ | ● | ○ | essential |

Clear selection

Does your paper address subitem 1b-iii?

Copy and paste relevant sections from the manuscript abstract (include quotes in quotation marks "like this" to indicate direct quotes from your manuscript), or elaborate on this item by providing additional information not in the ms, or briefly explain why the item is not applicable/relevant for your study

The combined exercise group engaged in unsupervised aerobic exercise (30 min, 3x/week), supervised group-based resistance training (30 min, 2x/week via Zoom), and one recorded class weekly, supplemented with four biweekly behavioral counseling sessions. The active control group participated in low-intensity balance and flexibility classes (30 min, 2x/week live, 1x/week recorded). Feasibility was assessed via enrollment, adherence, attrition, measurement completion, adverse events, and participant satisfaction. Cognitive function was evaluated using the NIH Toolbox Cognitive Battery Remote Administration (V2) at baseline and post-intervention.

1b-iv) RESULTS section in abstract must contain use data

Report number of participants enrolled/assessed in each group, the use/uptake of the intervention (e.g., attrition/adherence metrics, use over time, number of logins etc.), in addition to primary/secondary outcomes. (Note: Only report in the abstract what the main paper is reporting. If this information is missing from the main body of text, consider adding it)

| | 1 | 2 | 3 | 4 | 5 | |
|---|---|---|---|---|---|---|
| subitem not at all important | ○ | ○ | ○ | ○ | ● | essential |

Clear selection

Does your paper address subitem 1b-iv?

Copy and paste relevant sections from the manuscript abstract (include quotes in quotation marks "like this" to indicate direct quotes from your manuscript), or elaborate on this item by providing additional information not in the ms, or briefly explain why the item is not applicable/relevant for your study

A total of 21 participants (Mage=51.6±7.2 years; mean months since treatment=11.8±12.9) were randomized to the combined exercise group (n=10) or the active control group (n=11). Final analyses included 18 participants (Mage=51.9±7.4 years; mean months since treatment=12.6±13.5) with nine participants in each group. The study achieved a 51.2% enrollment rate, with a 14.3% attrition rate and no adverse events. Measurement completion rates were 85.7%, and participants reported high satisfaction with the intervention, indicating minimal burden. Adherence rates for exercise classes for both groups were 70.8%. Adherence to the behavioral counseling sessions was 77.5% that was delivered to the combined exercise group only. There were no significant statistical differences in objectively-measured cognitive function, but small to medium effect size improvements were observed in episodic memory (MDiff=+5.33, 95% CI: -12.5–23.2, $\eta p^2$=0.03), working memory (MDiff=+8.17, 95% CI=-4.2–20.6, $\eta p^2$=0.12), executive function updating (MDiff=-394.35ms, 95% CI: -1035.67–246.96, $\eta p^2$=0.07), and immediate memory and verbal learning (MDiff=+3.22, 95% CI: -2.0–8.5, $\eta p^2$=0.12), favoring the multicomponent exercise group versus the active control group. In contrast, the active control group demonstrated greater improvements in the Oral Reading Recognition Test (MDiff=-9.65, 95% CI: -22.9-3.5, p=0.14, $\eta p^2$=0.14) and Picture Vocabulary Test (MDiff=-2.48, 95% CI: -5.2–0.3, p=0.07, $\eta p^2$=0.20).

1b-v) CONCLUSIONS/DISCUSSION in abstract for negative trials

Conclusions/Discussions in abstract for negative trials: Discuss the primary outcome - if the trial is negative (primary outcome not changed), and the intervention was not used, discuss whether negative results are attributable to lack of uptake and discuss reasons. (Note: Only report in the abstract what the main paper is reporting. If this information is missing from the main body of text, consider adding it)

| | 1 | 2 | 3 | 4 | 5 | |
|---|---|---|---|---|---|---|
| subitem not at all important | ○ | ○ | ○ | ◉ | ○ | essential |

Clear selection

Does your paper address subitem 1b-v?

Copy and paste relevant sections from the manuscript abstract (include quotes in quotation marks "like this" to indicate direct quotes from your manuscript), or elaborate on this item by providing additional information not in the ms, or briefly explain why the item is not applicable/relevant for your study

A remotely-delivered combined exercise intervention is feasible and holds promise for improving CRCI in BC patients. Larger randomized controlled trials are warranted to confirm its efficacy in enhancing cognitive function and QoL in this population.

INTRODUCTION

2a) In INTRODUCTION: Scientific background and explanation of rationale

2a-i) Problem and the type of system/solution

Describe the problem and the type of system/solution that is object of the study: intended as stand-alone intervention vs. incorporated in broader health care program? Intended for a particular patient population? Goals of the intervention, e.g., being more cost-effective to other interventions, replace or complement other solutions? (Note: Details about the intervention are provided in “Methods” under 5)

| | 1 | 2 | 3 | 4 | 5 | |
|---|---|---|---|---|---|---|
| subitem not at all important | ○ | ○ | ○ | ○ | ◉ | essential |

Clear selection

Does your paper address subitem 2a-i? *

Copy and paste relevant sections from the manuscript (include quotes in quotation marks "like this" to indicate direct quotes from your manuscript), or elaborate on this item by providing additional information not in the ms, or briefly explain why the item is not applicable/relevant for your study

An increasing number of breast cancer (BC) patients are experiencing chronic symptoms due to rising cancer incidence and improving survival rates [1]. Treatments such as chemotherapy, radiation therapy, and hormone therapy while effective at reducing mortality, are associated with many adverse side effects including cognitive decline known as cancer-related cognitive impairment (CRCI) [2]. CRCI has been reported in up to 85% of BC patients, lasting for months, years, and not uncommonly indefinitely following treatment completion [3]. CRCI manifests as problems with attention, processing speed, memory, and executive function, most commonly reported following chemotherapy [4-6]. Chemotherapeutic drugs may have neurotoxic effects, leading to structural and functional changes in the brain [4, 7-9]. Other chemotherapy-related side-effects such as fatigue, anxiety, depression, stress, and sleep dysfunction may further degrade cognition in BC patients [11, 12]. These cognitive problems can significantly impair work performance, social relationships, and daily functioning, ultimately reducing quality of life (QoL) [13, 14]. Despite the prevalence and significance of this problem, as yet no established treatment has been identified to mitigate the adverse effects of CRCI on BC patients [15].

2a-ii) Scientific background, rationale: What is known about the (type of) system

Scientific background, rationale: What is known about the (type of) system that is the object of the study (be sure to discuss the use of similar systems for other conditions/diagnoses, if appropiate), motivation for the study, i.e. what are the reasons for and what is the context for this specific study, from which stakeholder viewpoint is the study performed, potential impact of findings [2]. Briefly justify the choice of the comparator.

| | 1 | 2 | 3 | 4 | 5 | |
|---|---|---|---|---|---|---|
| subitem not at all important | ○ | ○ | ○ | ○ | ◉ | essential |

Clear selection

Does your paper address subitem 2a-ii? *

Copy and paste relevant sections from the manuscript (include quotes in quotation marks "like this" to indicate direct quotes from your manuscript), or elaborate on this item by providing additional information not in the ms, or briefly explain why the item is not applicable/relevant for your study

Despite growing interest in this area, findings are preliminary, with limited evidence derived from high-quality powered randomized controlled trials (RCT) in BC patients [3, 18]. For example, a three-arm RCT compared 16 weeks of concurrent aerobic-resistance exercise to continuous moderate-intensity aerobic exercise with high-intensity interval training and usual care in 206 BC patients following chemotherapy. Immediately post-intervention, the aerobic-resistance training group had no changes in self-reported cognitive function. Whereas lower cognitive cancer-related fatigue was noted at the two-year follow-up in this group [21, 22]. Nevertheless, the lack of data on the specific type and frequency of exercise during the follow-up period makes it challenging to attribute these improvements solely to the initial aerobic-resistance exercise intervention, particularly concerning its impact on objectively measured cognitive function. Key gaps were noted in the literature regarding exercise interventions, including a limited understanding of the required exercise dose to elicit cognitive benefits, and insufficient attention to behaviour change support necessary for exercise maintenance to sustain exercise engagement and reductions in long-term CRCI. Moreover, inconsistent inclusion of BC patients with self-reported CRCI following chemotherapy, absence of active comparators, and considerable heterogeneity in cognitive function measures are also notable gaps in the literature. As such, the available evidence is insufficient for expert consensus to guide exercise recommendations for CRCI management. Additional research is warranted.

2b) In INTRODUCTION: Specific objectives or hypotheses

Does your paper address CONSORT subitem 2b? *

Copy and paste relevant sections from the manuscript (include quotes in quotation marks "like this" to indicate direct quotes from your manuscript), or elaborate on this item by providing additional information not in the ms, or briefly explain why the item is not applicable/relevant for your study

The primary purpose of this study was to assess the feasibility of an 8-week, remotely-delivered, combined exercise intervention (aerobic + resistance training) on cognitive function in BC patients following chemotherapy. Feasibility indicators included enrolment rates, adherence (defined as maintaining an exercise rate of [illegible]70%), attrition rates (<30%), adverse events, participant satisfaction, and feedback. It was hypothesized that the trial would meet the following a priori feasibility indicators: adherence rate 70%, attrition rate <30% [31-33], and that no adverse events or major injuries would occur as a result of the combined exercise intervention. We also predicted that the combined exercise group would result in significantly greater improvements in objective measures of attention, executive function, and memory (secondary outcomes) compared to the active control group at post-intervention (8 weeks), with sustained exercise.

METHODS

3a) Description of trial design (such as parallel, factorial) including allocation ratio

Does your paper address CONSORT subitem 3a? *

Copy and paste relevant sections from the manuscript (include quotes in quotation marks "like this" to indicate direct quotes from your manuscript), or elaborate on this item by providing additional information not in the ms, or briefly explain why the item is not applicable/relevant for your study

This two-armed, parallel-design pilot RCT evaluated the effects of an 8-week, remotely-delivered combined (aerobic + resistance training) exercise program compared to an active control group, with participants randomized in a 1:1 allocation ratio among inactive BC patients following chemotherapy.

3b) Important changes to methods after trial commencement (such as eligibility criteria), with reasons

Does your paper address CONSORT subitem 3b? *

Copy and paste relevant sections from the manuscript (include quotes in quotation marks "like this" to indicate direct quotes from your manuscript), or elaborate on this item by providing additional information not in the ms, or briefly explain why the item is not applicable/relevant for your study

not applicable as intervention was not an app.

3b-i) Bug fixes, Downtimes, Content Changes

Bug fixes, Downtimes, Content Changes: ehealth systems are often dynamic systems. A description of changes to methods therefore also includes important changes made on the intervention or comparator during the trial (e.g., major bug fixes or changes in the functionality or content) (5-iii) and other “unexpected events” that may have influenced study design such as staff changes, system failures/downtimes, etc. [2].

| | 1 | 2 | 3 | 4 | 5 | |
|---|---|---|---|---|---|---|
| subitem not at all important | (x) | ( ) | ( ) | ( ) | ( ) | essential |

Clear selection

Does your paper address subitem 3b-i?

Copy and paste relevant sections from the manuscript (include quotes in quotation marks "like this" to indicate direct quotes from your manuscript), or elaborate on this item by providing additional information not in the ms, or briefly explain why the item is not applicable/relevant for your study

not applicable as intervention was not an app.

4a) Eligibility criteria for participants

Does your paper address CONSORT subitem 4a? *

Copy and paste relevant sections from the manuscript (include quotes in quotation marks "like this" to indicate direct quotes from your manuscript), or elaborate on this item by providing additional information not in the ms, or briefly explain why the item is not applicable/relevant for your study

Eligibility criteria included: a) 40-65 years of age; b) a history of stage I-III BC (non-metastatic); c) a history of receiving chemotherapy within 48 months prior to enrollment; d) mild cognitive impairment as determined by the Telephone Interview of Cognitive Status (TICS-M) [scores between 21-24 to separate individuals with mild cognitive impairment and normal cognition (>24)] (required for the cognitive function outcome assessed in the primary study [35]; e) self-reported low-active defined as <3 days of exercise (<20 min/day) per week in the previous six months [36]; f) physician clearance to participate in exercise if required; g) no previous invasive cancer; h) no neurological or musculoskeletal co-morbidity inhibiting exercise; i) access to a webcam and internet for videoconferencing; and j) English fluency.

4a-i) Computer / Internet literacy

Computer / Internet literacy is often an implicit "de facto" eligibility criterion - this should be explicitly clarified.

| | 1 | 2 | 3 | 4 | 5 | |
|---|---|---|---|---|---|---|
| subitem not at all important | ○ | ○ | ○ | ◉ | ○ | essential |

Clear selection

Does your paper address subitem 4a-i?

Copy and paste relevant sections from the manuscript (include quotes in quotation marks "like this" to indicate direct quotes from your manuscript), or elaborate on this item by providing additional information not in the ms, or briefly explain why the item is not applicable/relevant for your study

Eligibility criteria included: a) 40-65 years of age; b) a history of stage I-III BC (non-metastatic); c) a history of receiving chemotherapy within 48 months prior to enrollment; d) mild cognitive impairment as determined by the Telephone Interview of Cognitive Status (TICS-M) [scores between 21-24 to separate individuals with mild cognitive impairment and normal cognition (>24)] (required for the cognitive function outcome assessed in the primary study [35]; e) self-reported low-active defined as <3 days of exercise (<20 min/day) per week in the previous six months [36]; f) physician clearance to participate in exercise if required; g) no previous invasive cancer; h) no neurological or musculoskeletal co-morbidity inhibiting exercise; i) access to a webcam and internet for videoconferencing; and j) English fluency.

4a-ii) Open vs. closed, web-based vs. face-to-face assessments:

Open vs. closed, web-based vs. face-to-face assessments: Mention how participants were recruited (online vs. offline), e.g., from an open access website or from a clinic, and clarify if this was a purely web-based trial, or there were face-to-face components (as part of the intervention or for assessment), i.e., to what degree got the study team to know the participant. In online-only trials, clarify if participants were quasi-anonymous and whether having multiple identities was possible or whether technical or logistical measures (e.g., cookies, email confirmation, phone calls) were used to detect/prevent these.

| | 1 | 2 | 3 | 4 | 5 | |
|---|---|---|---|---|---|---|
| subitem not at all important | ○ | ○ | ○ | ● | ○ | essential |

Clear selection

Does your paper address subitem 4a-ii? *

Copy and paste relevant sections from the manuscript (include quotes in quotation marks "like this" to indicate direct quotes from your manuscript), or elaborate on this item by providing additional information not in the ms, or briefly explain why the item is not applicable/relevant for your study

Participants were recruited from cancer care organizations, support groups, university listservs, existing database of research participants who participated in prior research studies in the lab, and social media advertisements (i.e., Facebook, Instagram, X) across Canada between February and July 2023.

4a-iii) Information giving during recruitment

Information given during recruitment. Specify how participants were briefed for recruitment and in the informed consent procedures (e.g., publish the informed consent documentation as appendix, see also item X26), as this information may have an effect on user self-selection, user expectation and may also bias results.

| | 1 | 2 | 3 | 4 | 5 | |
|---|---|---|---|---|---|---|
| subitem not at all important | ○ | ○ | ○ | ◉ | ○ | essential |

Clear selection

Does your paper address subitem 4a-iii?

Copy and paste relevant sections from the manuscript (include quotes in quotation marks "like this" to indicate direct quotes from your manuscript), or elaborate on this item by providing additional information not in the ms, or briefly explain why the item is not applicable/relevant for your study

Eligible participants were randomized in a 1:1 ratio to either the combined exercise group or the active control group using Research Electronic Data Capture (REDCap) [37, 38]. Randomization was conducted upon completion of the informed consent form and all baseline measures (i.e., physical activity (PA) assessment and a battery of questionnaires). Participants were blinded to the study hypotheses and were only informed of their assigned group. Outcome assessors were kept blinded to the allocation.

4b) Settings and locations where the data were collected

Does your paper address CONSORT subitem 4b? *

Copy and paste relevant sections from the manuscript (include quotes in quotation marks "like this" to indicate direct quotes from your manuscript), or elaborate on this item by providing additional information not in the ms, or briefly explain why the item is not applicable/relevant for your study

Participants in both groups participated in an 8-week, remotely delivered, supervised exercise program conducted via videoconferencing (Zoom). The program consisted of two 30-minute live sessions and one 30-minute recorded session per week (Figure 1). Recorded sessions aligned with live classes to maintain consistency and allowed for individualized adjustments. All live sessions were led by Qualified Exercise Professionals (QEPs) certified as registered Kinesiologists (RKins) or through the American College of Sports Medicine (ACSM) or Canadian Society of Exercise Physiologists (CSEP). Participants were encouraged to keep their cameras on during live sessions to facilitate safety monitoring and provide real-time exercise modifications.

4b-i) Report if outcomes were (self-)assessed through online questionnaires

Clearly report if outcomes were (self-)assessed through online questionnaires (as common in web-based trials) or otherwise.

| | 1 | 2 | 3 | 4 | 5 | |
|---|---|---|---|---|---|---|
| subitem not at all important | ○ | ○ | ○ | ○ | ◉ | essential |

Clear selection

Does your paper address subitem 4b-i? *

Copy and paste relevant sections from the manuscript (include quotes in quotation marks "like this" to indicate direct quotes from your manuscript), or elaborate on this item by providing additional information not in the ms, or briefly explain why the item is not applicable/relevant for your study

Feasibility was assessed by evaluating the enrolment, adherence (i.e., attendance and exercise prescription adherence), attrition rates, measurement completion rates, adverse events, program satisfaction, and therapeutic alliance with the QEP. The enrollment rate was determined by the percentage of participants assessed for eligibility who subsequently enrolled. Attendance was expressed as a percentage of exercise sessions attended, and exercise prescription adherence was determined by assessing self-reported RPE and Fitbit Inspire 2 measured HR during each exercise session. Attrition was measured as the percentage of participants who did not complete the intervention. Participants completed a patient satisfaction and therapeutic alliance questionnaire following the intervention (i.e., 8-weeks). The Working Alliance Inventory Short Revised (WAI-SR) [43, 44], with higher scores representing better therapeutic alliance. Closed-ended questions with responses options on a Likert 7-point scale ranging from 1 (not at all) to 7 (very much) were used.

Cognitive function was assessed using the remote National Institutes of Health (NIH) Toolbox Cognitive Battery (v2), a validated tool with minimal practice effects comparable to other widely used gold-standard cognitive measures [45,46]. The NIH Toolbox Battery included four tests: the Picture Vocabulary Test, which assessed language and vocabulary knowledge; the Picture Sequence Memory Test, which measured episodic memory; the List Sort Working Memory Test, which evaluated working memory and executive function updating; and the Auditory Verbal Learning Test, which assessed immediate memory and verbal learning [45,46].

As the NIH Toolbox does not include a measure of executive function shifting - a domain frequently impaired in BC survivors treated with chemotherapy [15,47] - the PsyToolkit Task Switch cognitive test was administered as a supplementary measure [48, 49]. The NIH Toolbox Cognition Battery was administered remotely at both time points via a shared iPad (9.7” iPad 2; Apple, CA) screen over Zoom in a quiet, distraction-free room, with each session taking approximately 45 minutes to complete. The examiner completed the remote administration training protocol (https://nihtoolbox.zendesk.com/hc/en-us/articles/13434920124820-Remote-Administration-in-NIH-Toolbox-V2) and operated under the guidance of researchers with expertise in cognitive psychology (AFK, JR). A single examiner administered the battery via a shared screen ensure that the participant was in unison with the examiner while navigating through the test. The examiner was able to see both the tablet screen and participant. This approach ensured standardized and objective cognitive assessment across participants.
The NIH Toolbox Battery was administered remotely at both time points via a shared iPad screen over Zoom, with each session taking approximately 45 minutes to complete. This approach ensured standardized and objective cognitive assessment across participants.

Device-based Exercise Minutes was measured with Actigraph GTX3+ accelerometers (Pensacola, FL). Participants wore the accelerometer on their non-dominant hip during waking hours for 7 consecutive days. Data was analyzed if there were no extreme counts (> 20,000) and if data were available for at least 10 valid hours of wear time on 4 or more days. Data was downloaded in 60-second epochs and then processed and converted to mean counts per minute in the ActiLife software package (v6.13.5) to estimate daily minutes of light (101–1951 counts $min^{-1}$), moderate (1952–5724 counts $min^{-1}$), vigorous (≥ 5725 counts $min^{-1}$), and total moderate-to-vigorous intensity exercise (≥ 1952 counts $min^{-1}$) based on established cut-points [50]. Further, weekly minutes spent in each exercise [illegible]

intensity category was calculated and presented.

Demographic and clinical information were collected at baseline using a standardized health history questionnaire via self-report used in previous studies [51,52,53]. Demographic variables included age, marital status, education, employment, ethnicity, smoking, and alcohol use. Clinical information included months since diagnosis, months since treatment, type of cancer treatment received, current cancer status, and general health status. Additional data on comorbidities such as high blood pressure, high cholesterol, diabetes, and arthritis, were also obtained.

4b-ii) Report how institutional affiliations are displayed

Report how institutional affiliations are displayed to potential participants [on ehealth media], as affiliations with prestigious hospitals or universities may affect volunteer rates, use, and reactions with regards to an intervention.(Not a required item – describe only if this may bias results)

| | 1 | 2 | 3 | 4 | 5 | |
|---|---|---|---|---|---|---|
| subitem not at all important | ○ | ○ | ◉ | ○ | ○ | essential |

Clear selection

Does your paper address subitem 4b-ii?

Copy and paste relevant sections from the manuscript (include quotes in quotation marks "like this" to indicate direct quotes from your manuscript), or elaborate on this item by providing additional information not in the ms, or briefly explain why the item is not applicable/relevant for your study

institutional affiliation are displayed on the consent form as required by the research ethics board

5) The interventions for each group with sufficient details to allow replication, including how and when they were actually administered

### 5-i) Mention names, credential, affiliations of the developers, sponsors, and owners

Mention names, credential, affiliations of the developers, sponsors, and owners [6] (if authors/evaluators are owners or developer of the software, this needs to be declared in a “Conflict of interest” section or mentioned elsewhere in the manuscript).

| | 1 | 2 | 3 | 4 | 5 | |
|---|---|---|---|---|---|---|
| subitem not at all important | ○ | ○ | ○ | ◉ | ○ | essential |

Clear selection

### Does your paper address subitem 5-i?

Copy and paste relevant sections from the manuscript (include quotes in quotation marks "like this" to indicate direct quotes from your manuscript), or elaborate on this item by providing additional information not in the ms, or briefly explain why the item is not applicable/relevant for your study

Participants in both groups participated in an 8-week, remotely delivered, supervised exercise program conducted via videoconferencing (Zoom). The program consisted of two 30-minute live sessions and one 30-minute recorded session per week (Figure 1). Recorded sessions aligned with live classes to maintain consistency and allowed for individualized adjustments. All live sessions were led by Qualified Exercise Professionals (QEPs) certified as registered Kinesiologists (RKins) or through the American College of Sports Medicine (ACSM) or Canadian Society of Exercise Physiologists (CSEP). Participants were encouraged to keep their cameras on during live sessions to facilitate safety monitoring and provide real-time exercise modifications.

### 5-ii) Describe the history/development process

Describe the history/development process of the application and previous formative evaluations (e.g., focus groups, usability testing), as these will have an impact on adoption/use rates and help with interpreting results.

| | 1 | 2 | 3 | 4 | 5 | |
|---|---|---|---|---|---|---|
| subitem not at all important | ○ | ○ | ○ | ◉ | ○ | essential |

Clear selection

Does your paper address subitem 5-ii?

Copy and paste relevant sections from the manuscript (include quotes in quotation marks "like this" to indicate direct quotes from your manuscript), or elaborate on this item by providing additional information not in the ms, or briefly explain why the item is not applicable/relevant for your study

not applicable as this is a feasibility/pilot study

5-iii) Revisions and updating

Revisions and updating. Clearly mention the date and/or version number of the application/intervention (and comparator, if applicable) evaluated, or describe whether the intervention underwent major changes during the evaluation process, or whether the development and/or content was “frozen” during the trial. Describe dynamic components such as news feeds or changing content which may have an impact on the replicability of the intervention (for unexpected events see item 3b).

| | 1 | 2 | 3 | 4 | 5 | |
|---|---|---|---|---|---|---|
| subitem not at all important | ○ | ○ | ● | ○ | ○ | essential |

Clear selection

Does your paper address subitem 5-iii?

Copy and paste relevant sections from the manuscript (include quotes in quotation marks "like this" to indicate direct quotes from your manuscript), or elaborate on this item by providing additional information not in the ms, or briefly explain why the item is not applicable/relevant for your study

not applicable as this is a feasibility/pilot study

5-iv) Quality assurance methods

Provide information on quality assurance methods to ensure accuracy and quality of information provided [1], if applicable.

| | 1 | 2 | 3 | 4 | 5 | |
|---|---|---|---|---|---|---|
| subitem not at all important | ○ | ○ | ○ | ◉ | ○ | essential |

Clear selection

Does your paper address subitem 5-iv?

Copy and paste relevant sections from the manuscript (include quotes in quotation marks "like this" to indicate direct quotes from your manuscript), or elaborate on this item by providing additional information not in the ms, or briefly explain why the item is not applicable/relevant for your study

Participants in both groups participated in an 8-week, remotely delivered, supervised exercise program conducted via videoconferencing (Zoom). The program consisted of two 30-minute live sessions and one 30-minute recorded session per week (Figure 1). Recorded sessions aligned with live classes to maintain consistency and allowed for individualized adjustments. All live sessions were led by Qualified Exercise Professionals (QEPs) certified as registered Kinesiologists (RKins) or through the American College of Sports Medicine (ACSM) or Canadian Society of Exercise Physiologists (CSEP). Participants were encouraged to keep their cameras on during live sessions to facilitate safety monitoring and provide real-time exercise modifications.

5-v) Ensure replicability by publishing the source code, and/or providing screenshots/screen-capture video, and/or providing flowcharts of the algorithms used

Ensure replicability by publishing the source code, and/or providing screenshots/screen-capture video, and/or providing flowcharts of the algorithms used. Replicability (i.e., other researchers should in principle be able to replicate the study) is a hallmark of scientific reporting.

| | 1 | 2 | 3 | 4 | 5 | |
|---|---|---|---|---|---|---|
| subitem not at all important | ○ | ○ | ○ | ○ | ○ | essential |

Does your paper address subitem 5-v?

Copy and paste relevant sections from the manuscript (include quotes in quotation marks "like this" to indicate direct quotes from your manuscript), or elaborate on this item by providing additional information not in the ms, or briefly explain why the item is not applicable/relevant for your study

not applicable as this is not an app

5-vi) Digital preservation

Digital preservation: Provide the URL of the application, but as the intervention is likely to change or disappear over the course of the years; also make sure the intervention is archived (Internet Archive, webcitation.org, and/or publishing the source code or screenshots/videos alongside the article). As pages behind login screens cannot be archived, consider creating demo pages which are accessible without login.

| | 1 | 2 | 3 | 4 | 5 | |
|---|---|---|---|---|---|---|
| subitem not at all important | ○ | ○ | ● | ○ | ○ | essential |

Clear selection

Does your paper address subitem 5-vi?

Copy and paste relevant sections from the manuscript (include quotes in quotation marks "like this" to indicate direct quotes from your manuscript), or elaborate on this item by providing additional information not in the ms, or briefly explain why the item is not applicable/relevant for your study

not applicable as this is not an app

5-vii) Access

Access: Describe how participants accessed the application, in what setting/context, if they had to pay (or were paid) or not, whether they had to be a member of specific group. If known, describe how participants obtained "access to the platform and Internet" [1]. To ensure access for editors/reviewers/readers, consider to provide a "backdoor" login account or demo mode for reviewers/readers to explore the application (also important for archiving purposes, see vi).

| | 1 | 2 | 3 | 4 | 5 | |
|---|---|---|---|---|---|---|
| subitem not at all important | ○ | ○ | ○ | ○ | ◉ | essential |

Clear selection

Does your paper address subitem 5-vii? *

Copy and paste relevant sections from the manuscript (include quotes in quotation marks "like this" to indicate direct quotes from your manuscript), or elaborate on this item by providing additional information not in the ms, or briefly explain why the item is not applicable/relevant for your study

Participants in both groups participated in an 8-week, remotely delivered, supervised exercise program conducted via videoconferencing (Zoom). The program consisted of two 30-minute live sessions and one 30-minute recorded session per week (Figure 1). Recorded sessions aligned with live classes to maintain consistency and allowed for individualized adjustments. All live sessions were led by Qualified Exercise Professionals (QEPs) certified as registered Kinesiologists (RKins) or through the American College of Sports Medicine (ACSM) or Canadian Society of Exercise Physiologists (CSEP). Participants were encouraged to keep their cameras on during live sessions to facilitate safety monitoring and provide real-time exercise modifications.

### 5-viii) Mode of delivery, features/functionalities/components of the intervention and comparator, and the theoretical framework

Describe mode of delivery, features/functionalities/components of the intervention and comparator, and the theoretical framework [6] used to design them (instructional strategy [1], behaviour change techniques, persuasive features, etc., see e.g., [7, 8] for terminology). This includes an in-depth description of the content (including where it is coming from and who developed it) [1],” whether [and how] it is tailored to individual circumstances and allows users to track their progress and receive feedback” [6]. This also includes a description of communication delivery channels and – if computer-mediated communication is a component – whether communication was synchronous or asynchronous [6]. It also includes information on presentation strategies [1], including page design principles, average amount of text on pages, presence of hyperlinks to other resources, etc. [1].

| | 1 | 2 | 3 | 4 | 5 | |
|---|---|---|---|---|---|---|
| subitem not at all important | ○ | ○ | ○ | ○ | ◉ | essential |

Clear selection

Does your paper address subitem 5-viii? *

Copy and paste relevant sections from the manuscript (include quotes in quotation marks "like this" to indicate direct quotes from your manuscript), or elaborate on this item by providing additional information not in the ms, or briefly explain why the item is not applicable/relevant for your study

Participants in both groups participated in an 8-week, remotely delivered, supervised exercise program conducted via videoconferencing (Zoom). The program consisted of two 30-minute live sessions and one 30-minute recorded session per week (Figure 1). Recorded sessions aligned with live classes to maintain consistency and allowed for individualized adjustments. All live sessions were led by Qualified Exercise Professionals (QEPs) certified as registered Kinesiologists (RKins) or through the American College of Sports Medicine (ACSM) or Canadian Society of Exercise Physiologists (CSEP). Participants were encouraged to keep their cameras on during live sessions to facilitate safety monitoring and provide real-time exercise modifications.

Combined Exercise Group: Participants received resistance bands, a Fitbit Inspire 2 HR monitor, and an exercise log. The program included 30 minutes of unsupervised aerobic exercise (e.g., walking) three times per week, gradually increasing intensity and duration from 40-59% of the maximum heart rate reserve initially to 60-70% by the end of the program. The program also included 30-minute supervised resistance training sessions two times per week focused on major muscle groups using 2–3 sets of 8–12 repetitions at a Rating of Perceived Exertion (RPE) of 12–15, progressively adjusted with stronger resistance bands. A 30-60 second rest was provided between sets and 60 seconds between exercises, during which the QEP demonstrated the next exercise and offered modifications. Participants recorded their Heart Rate (HR) and RPE in exercise logs after each session. Participants also attended four remotely-delivered, bi-weekly, 30-minute behavioral counselling sessions based on the Multi-Process Action Control (M-PAC) framework, emphasizing goal-setting, habit formation, and behavioral control to support long-term PA (Table 1) [39, 40].

Active Control Group: The control group participated in a low-intensity, whole-body stretching and toning program designed to improve balance and flexibility. Exercises targeted major muscle groups potentially affected by cancer treatments, such as steroid use, radiation therapy, or surgery [41]. Flexibility exercises, incorporating both static and dynamic movements, were introduced weekly to maintain engagement and progress using resistance bands. Intensity was maintained at a light level (RPE 9–10) [42]. Participants recorded their HR (using Fitbit devices) and RPE in exercise logs after each session.

5-ix) Describe use parameters

Describe use parameters (e.g., intended “doses” and optimal timing for use). Clarify what instructions or recommendations were given to the user, e.g., regarding timing, frequency, heaviness of use, if any, or was the intervention used ad libitum.

| | 1 | 2 | 3 | 4 | 5 | |
|---|---|---|---|---|---|---|
| subitem not at all important | ○ | ○ | ○ | ◉ | ○ | essential |

Clear selection

Does your paper address subitem 5-ix?

Copy and paste relevant sections from the manuscript (include quotes in quotation marks "like this" to indicate direct quotes from your manuscript), or elaborate on this item by providing additional information not in the ms, or briefly explain why the item is not applicable/relevant for your study

Combined Exercise Group: Participants received resistance bands, a Fitbit Inspire 2 HR monitor, and an exercise log. The program included 30 minutes of unsupervised aerobic exercise (e.g., walking) three times per week, gradually increasing intensity and duration from 40-59% of the maximum heart rate reserve initially to 60-70% by the end of the program. The program also included 30-minute supervised resistance training sessions two times per week focused on major muscle groups using 2–3 sets of 8–12 repetitions at a Rating of Perceived Exertion (RPE) of 12–15, progressively adjusted with stronger resistance bands. A 30-60 second rest was provided between sets and 60 seconds between exercises, during which the QEP demonstrated the next exercise and offered modifications. Participants recorded their Heart Rate (HR) and RPE in exercise logs after each session. Participants also attended four remotely-delivered, bi-weekly, 30-minute behavioral counselling sessions based on the Multi-Process Action Control (M-PAC) framework, emphasizing goal-setting, habit formation, and behavioral control to support long-term PA (Table 1) [39, 40].

Active Control Group: The control group participated in a low-intensity, whole-body stretching and toning program designed to improve balance and flexibility. Exercises targeted major muscle groups potentially affected by cancer treatments, such as steroid use, radiation therapy, or surgery [41]. Flexibility exercises, incorporating both static and dynamic movements, were introduced weekly to maintain engagement and progress using resistance bands. Intensity was maintained at a light level (RPE 9–10) [42]. Participants recorded their HR (using Fitbit devices) and RPE in exercise logs after each session.

### 5-x) Clarify the level of human involvement

Clarify the level of human involvement (care providers or health professionals, also technical assistance) in the e-intervention or as co-intervention (detail number and expertise of professionals involved, if any, as well as “type of assistance offered, the timing and frequency of the support, how it is initiated, and the medium by which the assistance is delivered”. It may be necessary to distinguish between the level of human involvement required for the trial, and the level of human involvement required for a routine application outside of a RCT setting (discuss under item 21 – generalizability).

| | 1 | 2 | 3 | 4 | 5 | |
|---|---|---|---|---|---|---|
| subitem not at all important | ○ | ○ | ○ | ◉ | ○ | essential |

Clear selection

Does your paper address subitem 5-x?

Copy and paste relevant sections from the manuscript (include quotes in quotation marks "like this" to indicate direct quotes from your manuscript), or elaborate on this item by providing additional information not in the ms, or briefly explain why the item is not applicable/relevant for your study

Participants in both groups participated in an 8-week, remotely delivered, supervised exercise program conducted via videoconferencing (Zoom). The program consisted of two 30-minute live sessions and one 30-minute recorded session per week (Figure 1). Recorded sessions aligned with live classes to maintain consistency and allowed for individualized adjustments. All live sessions were led by Qualified Exercise Professionals (QEPs) certified as registered Kinesiologists (RKins) or through the American College of Sports Medicine (ACSM) or Canadian Society of Exercise Physiologists (CSEP). Participants were encouraged to keep their cameras on during live sessions to facilitate safety monitoring and provide real-time exercise modifications.

Combined Exercise Group: Participants received resistance bands, a Fitbit Inspire 2 HR monitor, and an exercise log. The program included 30 minutes of unsupervised aerobic exercise (e.g., walking) three times per week, gradually increasing intensity and duration from 40-59% of the maximum heart rate reserve initially to 60-70% by the end of the program. The program also included 30-minute supervised resistance training sessions two times per week focused on major muscle groups using 2–3 sets of 8–12 repetitions at a Rating of Perceived Exertion (RPE) of 12–15, progressively adjusted with stronger resistance bands. A 30-60 second rest was provided between sets and 60 seconds between exercises, during which the QEP demonstrated the next exercise and offered modifications. Participants recorded their Heart Rate (HR) and RPE in exercise logs after each session. Participants also attended four remotely-delivered, bi-weekly, 30-minute behavioral counselling sessions based on the Multi-Process Action Control (M-PAC) framework, emphasizing goal-setting, habit formation, and behavioral control to support long-term PA (Table 1) [39, 40].

Active Control Group: The control group participated in a low-intensity, whole-body stretching and toning program designed to improve balance and flexibility. Exercises targeted major muscle groups potentially affected by cancer treatments, such as steroid use, radiation therapy, or surgery [41]. Flexibility exercises, incorporating both static and dynamic movements, were introduced weekly to maintain engagement and progress using resistance bands. Intensity was maintained at a light level (RPE 9–10) [42]. Participants recorded their HR (using Fitbit devices) and RPE in exercise logs after each session.

### 5-xi) Report any prompts/reminders used

Report any prompts/reminders used: Clarify if there were prompts (letters, emails, phone calls, SMS) to use the application, what triggered them, frequency etc. It may be necessary to distinguish between the level of prompts/reminders required for the trial, and the level of prompts/reminders for a routine application outside of a RCT setting (discuss under item 21 – generalizability).

| | 1 | 2 | 3 | 4 | 5 | |
|---|---|---|---|---|---|---|
| subitem not at all important | ○ | ○ | ○ | ○ | ◉ | essential |

Clear selection

Does your paper address subitem 5-xi? *

Copy and paste relevant sections from the manuscript (include quotes in quotation marks "like this" to indicate direct quotes from your manuscript), or elaborate on this item by providing additional information not in the ms, or briefly explain why the item is not applicable/relevant for your study

Participants in both groups participated in an 8-week, remotely delivered, supervised exercise program conducted via videoconferencing (Zoom). The program consisted of two 30-minute live sessions and one 30-minute recorded session per week (Figure 1). Recorded sessions aligned with live classes to maintain consistency and allowed for individualized adjustments. All live sessions were led by Qualified Exercise Professionals (QEPs) certified as registered Kinesiologists (RKins) or through the American College of Sports Medicine (ACSM) or Canadian Society of Exercise Physiologists (CSEP). Participants were encouraged to keep their cameras on during live sessions to facilitate safety monitoring and provide real-time exercise modifications.

Combined Exercise Group: Participants received resistance bands, a Fitbit Inspire 2 HR monitor, and an exercise log. The program included 30 minutes of unsupervised aerobic exercise (e.g., walking) three times per week, gradually increasing intensity and duration from 40-59% of the maximum heart rate reserve initially to 60-70% by the end of the program. The program also included 30-minute supervised resistance training sessions two times per week focused on major muscle groups using 2–3 sets of 8–12 repetitions at a Rating of Perceived Exertion (RPE) of 12–15, progressively adjusted with stronger resistance bands. A 30-60 second rest was provided between sets and 60 seconds between exercises, during which the QEP demonstrated the next exercise and offered modifications. Participants recorded their Heart Rate (HR) and RPE in exercise logs after each session. Participants also attended four remotely-delivered, bi-weekly, 30-minute behavioral counselling sessions based on the Multi-Process Action Control (M-PAC) framework, emphasizing goal-setting, habit formation, and behavioral control to support long-term PA (Table 1) [39, 40].

Active Control Group: The control group participated in a low-intensity, whole-body stretching and toning program designed to improve balance and flexibility. Exercises targeted major muscle groups potentially affected by cancer treatments, such as steroid use, radiation therapy, or surgery [41]. Flexibility exercises, incorporating both static and dynamic movements, were introduced weekly to maintain engagement and progress using resistance bands. Intensity was maintained at a light level (RPE 9–10) [42]. Participants recorded their HR (using Fitbit devices) and RPE in exercise logs after each session.

5-xii) Describe any co-interventions (incl. training/support)

Describe any co-interventions (incl. training/support): Clearly state any interventions that are provided in addition to the targeted eHealth intervention, as ehealth intervention may not be designed as stand-alone intervention. This includes training sessions and support [1]. It may be necessary to distinguish between the level of training required for the trial, and the level of training for a routine application outside of a RCT setting (discuss under item 21 – generalizability.

| | 1 | 2 | 3 | 4 | 5 | |
|---|---|---|---|---|---|---|
| subitem not at all important | ○ | ○ | ○ | ● | ○ | essential |

Clear selection

Does your paper address subitem 5-xii? *

Copy and paste relevant sections from the manuscript (include quotes in quotation marks "like this" to indicate direct quotes from your manuscript), or elaborate on this item by providing additional information not in the ms, or briefly explain why the item is not applicable/relevant for your study

not applicable and therefore not included

6a) Completely defined pre-specified primary and secondary outcome measures, including how and when they were assessed

Does your paper address CONSORT subitem 6a? *

Copy and paste relevant sections from the manuscript (include quotes in quotation marks "like this" to indicate direct quotes from your manuscript), or elaborate on this item by providing additional information not in the ms, or briefly explain why the item is not applicable/relevant for your study

Primary Outcome: Feasibility
Feasibility was assessed by evaluating the enrolment, adherence (i.e., attendance and exercise prescription adherence), attrition rates, measurement completion rates, adverse events, program satisfaction, and therapeutic alliance with the QEP. The enrollment rate was determined by the percentage of participants assessed for eligibility who subsequently enrolled. Attendance was expressed as a percentage of exercise sessions attended, and exercise prescription adherence was determined by assessing self-reported RPE and Fitbit Inspire 2 measured HR during each exercise session. Attrition was measured as the percentage of participants who did not complete the intervention. Participants completed a patient satisfaction and therapeutic alliance questionnaire following the intervention (i.e., 8-weeks). The Working Alliance Inventory Short Revised (WAI-SR) [43, 44], with higher scores representing better therapeutic alliance. Closed-ended questions with responses options on a Likert 7-point scale ranging from 1 (not at all) to 7 (very much) were used.

Secondary Outcomes
Cognitive function was assessed using the remote National Institutes of Health (NIH) Toolbox Cognitive Battery (v2), a validated tool with minimal practice effects comparable to other widely used gold-standard cognitive measures [45,46]. The NIH Toolbox Battery included four tests: the Picture Vocabulary Test, which assessed language and vocabulary knowledge; the Picture Sequence Memory Test, which measured episodic memory; the List Sort Working Memory Test, which evaluated working memory and executive function updating; and the Auditory Verbal Learning Test, which assessed immediate memory and verbal learning [45,46].

As the NIH Toolbox does not include a measure of executive function shifting - a domain frequently impaired in BC survivors treated with chemotherapy [15,47] - the PsyToolkit Task Switch cognitive test was administered as a supplementary measure [48, 49]. The NIH Toolbox Cognition Battery was administered remotely at both time points via a shared iPad (9.7" iPad 2; Apple, CA) screen over Zoom in a quiet, distraction-free room, with each session taking approximately 45 minutes to complete. The examiner completed the remote administration training protocol (https://nihtoolbox.zendesk.com/hc/en-us/articles/13434920124820-Remote-Administration-in-NIH-Toolbox-V2) and operated under the guidance of researchers with expertise in cognitive psychology (AFK, JR). A single examiner administered the battery via a shared screen ensure that the participant was in unison with the examiner while navigating through the test. The examiner was able to see both the tablet screen and participant. This approach ensured standardized and objective cognitive assessment across participants.

The NIH Toolbox Battery was administered remotely at both time points via a shared iPad screen over Zoom, with each session taking approximately 45 minutes to complete. This approach ensured standardized and objective cognitive assessment across participants. Device-based Exercise Minutes was measured with Actigraph GTX3+ accelerometers (Pensacola, FL). Participants wore the accelerometer on their non-dominant hip during waking hours for 7 consecutive days. Data was analyzed if there were no extreme counts (> 20,000) and if data were available for at least 10 valid hours of wear time on 4 or more days. Data was downloaded in 60-second epochs and then processed and converted to mean counts per minute in the ActiLife software package (v6.13.5) to estimate daily minutes of light (101–1951 counts $min^{-1}$), moderate (1952–5724 counts $min^{-1}$), vigorous ($\geq$ 5725 [illegible]

counts min−1), and total moderate-to-vigorous intensity exercise (≥ 1952 counts min−1) based on established cut-points [50]. Further, weekly minutes spent in each exercise intensity category was calculated and presented.

Demographic and clinical information were collected at baseline using a standardized health history questionnaire via self-report used in previous studies [51,52,53]. Demographic variables included age, marital status, education, employment, ethnicity, smoking, and alcohol use. Clinical information included months since diagnosis, months since treatment, type of cancer treatment received, current cancer status, and general health status. Additional data on comorbidities such as high blood pressure, high cholesterol, diabetes, and arthritis, were also obtained.

6a-i) Online questionnaires: describe if they were validated for online use and apply CHERRIES items to describe how the questionnaires were designed/deployed

If outcomes were obtained through online questionnaires, describe if they were validated for online use and apply CHERRIES items to describe how the questionnaires were designed/deployed [9].

| | 1 | 2 | 3 | 4 | 5 | |
|---|---|---|---|---|---|---|
| subitem not at all important | ○ | ○ | ○ | ◉ | ○ | essential |

Clear selection

Does your paper address subitem 6a-i?

Copy and paste relevant sections from manuscript text

Primary Outcome: Feasibility
Feasibility was assessed by evaluating the enrolment, adherence (i.e., attendance and exercise prescription adherence), attrition rates, measurement completion rates, adverse events, program satisfaction, and therapeutic alliance with the QEP. The enrollment rate was determined by the percentage of participants assessed for eligibility who subsequently enrolled. Attendance was expressed as a percentage of exercise sessions attended, and exercise prescription adherence was determined by assessing self-reported RPE and Fitbit Inspire 2 measured HR during each exercise session. Attrition was measured as the percentage of participants who did not complete the intervention. Participants completed a patient satisfaction and therapeutic alliance questionnaire following the intervention (i.e., 8-weeks). The Working Alliance Inventory Short Revised (WAI-SR) [43, 44], with higher scores representing better therapeutic alliance. Closed-ended questions with responses options on a Likert 7-point scale ranging from 1 (not at all) to 7 (very much) were used.

Demographic and clinical information were collected at baseline using a standardized health history questionnaire via self-report used in previous studies [51,52,53]. Demographic variables included age, marital status, education, employment, ethnicity, smoking, and alcohol use. Clinical information included months since diagnosis, months since treatment, type of cancer treatment received, current cancer status, and general health status. Additional data on comorbidities such as high blood pressure, high cholesterol, diabetes, and arthritis, were also obtained.

6a-ii) Describe whether and how “use” (including intensity of use/dosage) was defined/measured/monitored

Describe whether and how “use” (including intensity of use/dosage) was defined/measured/monitored (logins, logfile analysis, etc.). Use/adoption metrics are important process outcomes that should be reported in any ehealth trial.

| | 1 | 2 | 3 | 4 | 5 | |
|---|---|---|---|---|---|---|
| subitem not at all important | ○ | ○ | ○ | ◉ | ○ | essential |

Clear selection

Does your paper address subitem 6a-ii?

Copy and paste relevant sections from manuscript text

Participants in both groups participated in an 8-week, remotely delivered, supervised exercise program conducted via videoconferencing (Zoom). The program consisted of two 30-minute live sessions and one 30-minute recorded session per week (Figure 1). Recorded sessions aligned with live classes to maintain consistency and allowed for individualized adjustments. All live sessions were led by Qualified Exercise Professionals (QEPs) certified as registered Kinesiologists (RKins) or through the American College of Sports Medicine (ACSM) or Canadian Society of Exercise Physiologists (CSEP). Participants were encouraged to keep their cameras on during live sessions to facilitate safety monitoring and provide real-time exercise modifications.

Combined Exercise Group: Participants received resistance bands, a Fitbit Inspire 2 HR monitor, and an exercise log. The program included 30 minutes of unsupervised aerobic exercise (e.g., walking) three times per week, gradually increasing intensity and duration from 40-59% of the maximum heart rate reserve initially to 60-70% by the end of the program. The program also included 30-minute supervised resistance training sessions two times per week focused on major muscle groups using 2–3 sets of 8–12 repetitions at a Rating of Perceived Exertion (RPE) of 12–15, progressively adjusted with stronger resistance bands. A 30-60 second rest was provided between sets and 60 seconds between exercises, during which the QEP demonstrated the next exercise and offered modifications. Participants recorded their Heart Rate (HR) and RPE in exercise logs after each session. Participants also attended four remotely-delivered, bi-weekly, 30-minute behavioral counselling sessions based on the Multi-Process Action Control (M-PAC) framework, emphasizing goal-setting, habit formation, and behavioral control to support long-term PA (Table 1) [39, 40].

Active Control Group: The control group participated in a low-intensity, whole-body stretching and toning program designed to improve balance and flexibility. Exercises targeted major muscle groups potentially affected by cancer treatments, such as steroid use, radiation therapy, or surgery [41]. Flexibility exercises, incorporating both static and dynamic movements, were introduced weekly to maintain engagement and progress using resistance bands. Intensity was maintained at a light level (RPE 9–10) [42]. Participants recorded their HR (using Fitbit devices) and RPE in exercise logs after each session.

6a-iii) Describe whether, how, and when qualitative feedback from participants was obtained

Describe whether, how, and when qualitative feedback from participants was obtained (e.g., through emails, feedback forms, interviews, focus groups).

| | 1 | 2 | 3 | 4 | 5 | |
|---|---|---|---|---|---|---|
| subitem not at all important | ○ | ○ | ○ | ◉ | ○ | essential |

Clear selection

Does your paper address subitem 6a-iii?

Copy and paste relevant sections from manuscript text

no qualitative feedback was obtained

6b) Any changes to trial outcomes after the trial commenced, with reasons

Does your paper address CONSORT subitem 6b? *

Copy and paste relevant sections from the manuscript (include quotes in quotation marks "like this" to indicate direct quotes from your manuscript), or elaborate on this item by providing additional information not in the ms, or briefly explain why the item is not applicable/relevant for your study

no changes to trial outcomes after the trial commenced

7a) How sample size was determined

NPT: When applicable, details of whether and how the clustering by care provides or centers was addressed

7a-i) Describe whether and how expected attrition was taken into account when calculating the sample size

Describe whether and how expected attrition was taken into account when calculating the sample size.

| | 1 | 2 | 3 | 4 | 5 | |
|---|---|---|---|---|---|---|
| subitem not at all important | ○ | ○ | ○ | ◉ | ○ | essential |

Clear selection

Does your paper address subitem 7a-i?

Copy and paste relevant sections from manuscript title (include quotes in quotation marks "like this" to indicate direct quotes from your manuscript), or elaborate on this item by providing additional information not in the ms, or briefly explain why the item is not applicable/relevant for your study

An a priori power calculation was not performed as this was a feasibility study.

7b) When applicable, explanation of any interim analyses and stopping guidelines

Does your paper address CONSORT subitem 7b? *

Copy and paste relevant sections from the manuscript (include quotes in quotation marks "like this" to indicate direct quotes from your manuscript), or elaborate on this item by providing additional information not in the ms, or briefly explain why the item is not applicable/relevant for your study

not applicable as this was a feasibility/pilot study

8a) Method used to generate the random allocation sequence

NPT: When applicable, how care providers were allocated to each trial group

Does your paper address CONSORT subitem 8a? *

Copy and paste relevant sections from the manuscript (include quotes in quotation marks "like this" to indicate direct quotes from your manuscript), or elaborate on this item by providing additional information not in the ms, or briefly explain why the item is not applicable/relevant for your study

Eligible participants were randomized in a 1:1 ratio to either the combined exercise group or the active control group using Research Electronic Data Capture (REDCap) [37, 38]. Randomization was conducted upon completion of the informed consent form and all baseline measures (i.e., physical activity (PA) assessment and a battery of questionnaires).

8b) Type of randomisation; details of any restriction (such as blocking and block size)

Does your paper address CONSORT subitem 8b? *

Copy and paste relevant sections from the manuscript (include quotes in quotation marks "like this" to indicate direct quotes from your manuscript), or elaborate on this item by providing additional information not in the ms, or briefly explain why the item is not applicable/relevant for your study

Eligible participants were randomized in a 1:1 ratio to either the combined exercise group or the active control group using Research Electronic Data Capture (REDCap) [37, 38]. Randomization was conducted upon completion of the informed consent form and all baseline measures (i.e., physical activity (PA) assessment and a battery of questionnaires).

9) Mechanism used to implement the random allocation sequence (such as sequentially numbered containers), describing any steps taken to conceal the sequence until interventions were assigned

Does your paper address CONSORT subitem 9? *

Copy and paste relevant sections from the manuscript (include quotes in quotation marks "like this" to indicate direct quotes from your manuscript), or elaborate on this item by providing additional information not in the ms, or briefly explain why the item is not applicable/relevant for your study

Eligible participants were randomized in a 1:1 ratio to either the combined exercise group or the active control group using Research Electronic Data Capture (REDCap) [37, 38]. Randomization was conducted upon completion of the informed consent form and all baseline measures (i.e., physical activity (PA) assessment and a battery of questionnaires). Participants were blinded to the study hypotheses and were only informed of their assigned group. Outcome assessors were kept blinded to the allocation.

10) Who generated the random allocation sequence, who enrolled participants, and who assigned participants to interventions

Does your paper address CONSORT subitem 10? *

Copy and paste relevant sections from the manuscript (include quotes in quotation marks "like this" to indicate direct quotes from your manuscript), or elaborate on this item by providing additional information not in the ms, or briefly explain why the item is not applicable/relevant for your study

Eligible participants were randomized in a 1:1 ratio to either the combined exercise group or the active control group using Research Electronic Data Capture (REDCap) by the research assistant (NC) [37, 38]. Randomization was conducted upon completion of the informed consent form and all baseline measures (i.e., physical activity (PA) assessment and a battery of questionnaires). Participants were blinded to the study hypotheses and were only informed of their assigned group. Outcome assessors were kept blinded to the allocation.

11a) If done, who was blinded after assignment to interventions (for example, participants, care providers, those assessing outcomes) and how

NPT: Whether or not administering co-interventions were blinded to group assignment

11a-i) Specify who was blinded, and who wasn't

Specify who was blinded, and who wasn't. Usually, in web-based trials it is not possible to blind the participants [1, 3] (this should be clearly acknowledged), but it may be possible to blind outcome assessors, those doing data analysis or those administering co-interventions (if any).

| | 1 | 2 | 3 | 4 | 5 | |
|---|---|---|---|---|---|---|
| subitem not at all important | ○ | ○ | ○ | ○ | ◉ | essential |

Clear selection

Does your paper address subitem 11a-i? *

Copy and paste relevant sections from the manuscript (include quotes in quotation marks "like this" to indicate direct quotes from your manuscript), or elaborate on this item by providing additional information not in the ms, or briefly explain why the item is not applicable/relevant for your study

Participants were blinded to the study hypotheses and were only informed of their assigned group. Outcome assessors were kept blinded to the allocation.

11a-ii) Discuss e.g., whether participants knew which intervention was the "intervention of interest" and which one was the "comparator"

Informed consent procedures (4a-ii) can create biases and certain expectations - discuss e.g., whether participants knew which intervention was the "intervention of interest" and which one was the "comparator".

| | 1 | 2 | 3 | 4 | 5 | |
|---|---|---|---|---|---|---|
| subitem not at all important | ○ | ○ | ○ | ○ | ◉ | essential |

Clear selection

Does your paper address subitem 11a-ii?

Copy and paste relevant sections from the manuscript (include quotes in quotation marks "like this" to indicate direct quotes from your manuscript), or elaborate on this item by providing additional information not in the ms, or briefly explain why the item is not applicable/relevant for your study

Participants were blinded to the study hypotheses and were only informed of their assigned group. Outcome assessors were kept blinded to the allocation.

11b) If relevant, description of the similarity of interventions

(this item is usually not relevant for ehealth trials as it refers to similarity of a placebo or sham intervention to a active medication/intervention)

Does your paper address CONSORT subitem 11b? *

Copy and paste relevant sections from the manuscript (include quotes in quotation marks "like this" to indicate direct quotes from your manuscript), or elaborate on this item by providing additional information not in the ms, or briefly explain why the item is not applicable/relevant for your study

not applicable and therefore not included

12a) Statistical methods used to compare groups for primary and secondary outcomes

NPT: When applicable, details of whether and how the clustering by care providers or centers was addressed

Does your paper address CONSORT subitem 12a? *

Copy and paste relevant sections from the manuscript (include quotes in quotation marks "like this" to indicate direct quotes from your manuscript), or elaborate on this item by providing additional information not in the ms, or briefly explain why the item is not applicable/relevant for your study

Descriptive statistics (i.e., means, standard deviations) characterized the sample for demographic, clinical and feasibility outcomes. Z-scores were used to identify any outliers in the field. Analyses of Covariance (ANCOVA) were used to assess both primary and secondary outcomes of the study. The dependent variable was the mean difference of each intervention outcome (i.e., the difference between post-intervention and pre-intervention scores) while the independent variable was the group (i.e., combined exercise or active control). All analyses were conducted while controlling for the baseline values of each outcome. All analyses were conducted on an intention-to-treat basis. Given that the purpose of this feasibility trial was to inform a larger RCT, outcomes were interpreted for potential clinical significance based on the direction and magnitude of numerical differences. Partial eta squared ($\eta p2$) values were reported to describe the observed effect sizes.

12a-i) Imputation techniques to deal with attrition / missing values

Imputation techniques to deal with attrition / missing values: Not all participants will use the intervention/comparator as intended and attrition is typically high in ehealth trials. Specify how participants who did not use the application or dropped out from the trial were treated in the statistical analysis (a complete case analysis is strongly discouraged, and simple imputation techniques such as LOCF may also be problematic [4]).

| | 1 | 2 | 3 | 4 | 5 | |
|---|---|---|---|---|---|---|
| subitem not at all important | ○ | ○ | ○ | ○ | ◉ | essential |

Clear selection

Does your paper address subitem 12a-i? *

Copy and paste relevant sections from the manuscript (include quotes in quotation marks "like this" to indicate direct quotes from your manuscript), or elaborate on this item by providing additional information not in the ms, or briefly explain why the item is not applicable/relevant for your study

An intention-to-treat analysis was conducted and therefore adherence was calculated based on the attendance and included the dropouts (n=3), since they were enrolled and randomized at the start of the intervention. Consequently, the attrition rate was 14.3%. Consequently, the attrition rate was 14.3%. For the ANCOVA analyses, complete case analysis was applied (n=18).

12b) Methods for additional analyses, such as subgroup analyses and adjusted analyses

Does your paper address CONSORT subitem 12b? *

Copy and paste relevant sections from the manuscript (include quotes in quotation marks "like this" to indicate direct quotes from your manuscript), or elaborate on this item by providing additional information not in the ms, or briefly explain why the item is not applicable/relevant for your study

Descriptive statistics (i.e., means, standard deviations) characterized the sample for demographic, clinical and feasibility outcomes. Z-scores were used to identify any outliers in the field. Analyses of Covariance (ANCOVA) were used to assess both primary and secondary outcomes of the study. The dependent variable was the mean difference of each intervention outcome (i.e., the difference between post-intervention and pre-intervention scores) while the independent variable was the group (i.e., combined exercise or active control). All analyses were conducted while controlling for the baseline values of each outcome. All analyses were conducted on an intention-to-treat basis. Given that the purpose of this feasibility trial was to inform a larger RCT, outcomes were interpreted for potential clinical significance based on the direction and magnitude of numerical differences. Partial eta squared ($\eta_p^2$) values were reported to describe the observed effect sizes.

X26) REB/IRB Approval and Ethical Considerations [recommended as subheading under "Methods"] (not a CONSORT item)

X26-i) Comment on ethics committee approval

| | 1 | 2 | 3 | 4 | 5 | |
|---|---|---|---|---|---|---|
| subitem not at all important | ○ | ○ | ○ | ◉ | ○ | essential |

Clear selection

Does your paper address subitem X26-i?

Copy and paste relevant sections from the manuscript (include quotes in quotation marks "like this" to indicate direct quotes from your manuscript), or elaborate on this item by providing additional information not in the ms, or briefly explain why the item is not applicable/relevant for your study

The trial protocol was approved by the Research Ethics Board at the University of Toronto (#43675) and all participants provided written informed consent

x26-ii) Outline informed consent procedures

Outline informed consent procedures e.g., if consent was obtained offline or online (how? Checkbox, etc.?), and what information was provided (see 4a-ii). See [6] for some items to be included in informed consent documents.

| | 1 | 2 | 3 | 4 | 5 | |
|---|---|---|---|---|---|---|
| subitem not at all important | ○ | ○ | ○ | ◉ | ○ | essential |

Clear selection

Does your paper address subitem X26-ii?

Copy and paste relevant sections from the manuscript (include quotes in quotation marks "like this" to indicate direct quotes from your manuscript), or elaborate on this item by providing additional information not in the ms, or briefly explain why the item is not applicable/relevant for your study

Eligible participants were randomized in a 1:1 ratio to either the combined exercise group or the active control group using Research Electronic Data Capture (REDCap) [37, 38]. Randomization was conducted upon completion of the informed consent form and all baseline measures (i.e., physical activity (PA) assessment and a battery of questionnaires). Participants were blinded to the study hypotheses and were only informed of their assigned group. Outcome assessors were kept blinded to the allocation.

X26-iii) Safety and security procedures

Safety and security procedures, incl. privacy considerations, and any steps taken to reduce the likelihood or detection of harm (e.g., education and training, availability of a hotline)

| | 1 | 2 | 3 | 4 | 5 | |
|---|---|---|---|---|---|---|
| subitem not at all important | ○ | ○ | ○ | ◉ | ○ | essential |

Clear selection

Does your paper address subitem X26-iii?

Copy and paste relevant sections from the manuscript (include quotes in quotation marks "like this" to indicate direct quotes from your manuscript), or elaborate on this item by providing additional information not in the ms, or briefly explain why the item is not applicable/relevant for your study

Research Electronic Data Capture (REDCap) is a secure data collection tool that is used for self-reported questionnaire data and the consent process. Participants were also provided with a unique Zoom link with a passcode for the remotely-delivered intervention.

RESULTS

13a) For each group, the numbers of participants who were randomly assigned, received intended treatment, and were analysed for the primary outcome
NPT: The number of care providers or centers performing the intervention in each group and the number of patients treated by each care provider in each center

Does your paper address CONSORT subitem 13a? *

Copy and paste relevant sections from the manuscript (include quotes in quotation marks "like this" to indicate direct quotes from your manuscript), or elaborate on this item by providing additional information not in the ms, or briefly explain why the item is not applicable/relevant for your study

Participant flow through the study is presented in Figure 2. Of the 41 women who responded to the invitation, 22 were eligible and 21 consented to be randomized into either the combined exercise group (aerobic + resistance training; n=10) or the active control group (balance and flexibility; n=11), representing a 51.2% enrolment rate. Participants were divided into two cohorts: the first cohort (n=11) began in May 2023, and the second cohort (n=10) began in July 2023. The sample size aligns with previous research on feasibility and group cohesion for counselling programs for BCS [56,57]. Overall, 18 participants completed the 8-week intervention. Three participants withdrew after randomization, but before starting the intervention for reasons including a change in BC treatment plan (n=1), an issue with the timing of the programming and their schedule (n=1), and a change of interest in participating (n=1). An intention-to-treat analysis was conducted and therefore adherence was calculated based on the attendance and included the dropouts (n=3), since they were enrolled and randomized at the start of the intervention. Consequently, the attrition rate was 14.3%.

13b) For each group, losses and exclusions after randomisation, together with reasons

Does your paper address CONSORT subitem 13b? (NOTE: Preferably, this is shown in a CONSORT flow diagram) *

Copy and paste relevant sections from the manuscript (include quotes in quotation marks "like this" to indicate direct quotes from your manuscript), or elaborate on this item by providing additional information not in the ms, or briefly explain why the item is not applicable/relevant for your study

Participant flow through the study is presented in Figure 2. Of the 41 women who responded to the invitation, 22 were eligible and 21 consented to be randomized into either the combined exercise group (aerobic + resistance training; n=10) or the active control group (balance and flexibility; n=11), representing a 51.2% enrolment rate. Participants were divided into two cohorts: the first cohort (n=11) began in May 2023, and the second cohort (n=10) began in July 2023. The sample size aligns with previous research on feasibility and group cohesion for counselling programs for BCS [56,57]. Overall, 18 participants completed the 8-week intervention. Three participants withdrew after randomization, but before starting the intervention for reasons including a change in BC treatment plan (n=1), an issue with the timing of the programming and their schedule (n=1), and a change of interest in participating (n=1). An intention-to-treat analysis was conducted and therefore adherence was calculated based on the attendance and included the dropouts (n=3), since they were enrolled and randomized at the start of the intervention. Consequently, the attrition rate was 14.3%.

13b-i) Attrition diagram

Strongly recommended: An attrition diagram (e.g., proportion of participants still logging in or using the intervention/comparator in each group plotted over time, similar to a survival curve) or other figures or tables demonstrating usage/dose/engagement.

| | 1 | 2 | 3 | 4 | 5 | |
|---|---|---|---|---|---|---|
| subitem not at all important | ○ | ○ | ◉ | ○ | ○ | essential |

Clear selection

Does your paper address subitem 13b-i?

Copy and paste relevant sections from the manuscript or cite the figure number if applicable (include quotes in quotation marks "like this" to indicate direct quotes from your manuscript), or elaborate on this item by providing additional information not in the ms, or briefly explain why the item is not applicable/relevant for your study

see Figure 2 for flow diagram

14a) Dates defining the periods of recruitment and follow-up

Does your paper address CONSORT subitem 14a? *

Copy and paste relevant sections from the manuscript (include quotes in quotation marks "like this" to indicate direct quotes from your manuscript), or elaborate on this item by providing additional information not in the ms, or briefly explain why the item is not applicable/relevant for your study

Participants were recruited from cancer care organizations, support groups, university listservs, existing database of research participants who participated in prior research studies in the lab, and social media advertisements (i.e., Facebook, Instagram, X) across Canada between February and July 2023.

Primary outcomes of feasibility, demographic information, and participant satisfaction was assessed post-intervention. Secondary outcomes of PA and cognitive function were assessed at baseline and post-intervention.

14a-i) Indicate if critical “secular events” fell into the study period

Indicate if critical “secular events” fell into the study period, e.g., significant changes in Internet resources available or “changes in computer hardware or Internet delivery resources”

| | 1 | 2 | 3 | 4 | 5 | |
|---|---|---|---|---|---|---|
| subitem not at all important | ○ | ○ | ◉ | ○ | ○ | essential |

Clear selection

Does your paper address subitem 14a-i?

Copy and paste relevant sections from the manuscript (include quotes in quotation marks "like this" to indicate direct quotes from your manuscript), or elaborate on this item by providing additional information not in the ms, or briefly explain why the item is not applicable/relevant for your study

not applicable and therefore not included

14b) Why the trial ended or was stopped (early)

Does your paper address CONSORT subitem 14b? *

Copy and paste relevant sections from the manuscript (include quotes in quotation marks "like this" to indicate direct quotes from your manuscript), or elaborate on this item by providing additional information not in the ms, or briefly explain why the item is not applicable/relevant for your study

trial idd not stop or end early

15) A table showing baseline demographic and clinical characteristics for each group

NPT: When applicable, a description of care providers (case volume, qualification, expertise, etc.) and centers (volume) in each group

Does your paper address CONSORT subitem 15? *

Copy and paste relevant sections from the manuscript (include quotes in quotation marks "like this" to indicate direct quotes from your manuscript), or elaborate on this item by providing additional information not in the ms, or briefly explain why the item is not applicable/relevant for your study

See Table 2 for demographic and clinical characteristics

15-i) Report demographics associated with digital divide issues

In ehealth trials it is particularly important to report demographics associated with digital divide issues, such as age, education, gender, social-economic status, computer/Internet/ehealth literacy of the participants, if known.

| | 1 | 2 | 3 | 4 | 5 | |
|---|---|---|---|---|---|---|
| subitem not at all important | ○ | ○ | ○ | ○ | ◉ | essential |

Clear selection

Does your paper address subitem 15-i? *

Copy and paste relevant sections from the manuscript (include quotes in quotation marks "like this" to indicate direct quotes from your manuscript), or elaborate on this item by providing additional information not in the ms, or briefly explain why the item is not applicable/relevant for your study

Demographic and clinical characteristics of the participants are summarized in Table 2. Overall, all participants were female, identified as women (100%), predominantly White (76.2%), with an average age of 51.9 ± 7.2 years. The majority were married (81%), some had completed university or college (47.6%), and many were employed full-time (61.9%). All participants had chemotherapy and were 22.9 ± 13.9 months post-diagnosis and 11.8 ± 12.9 months post-treatment. Regarding weekly exercise minutes, participants engaged in 149.4 ± 96.1 moderate-to-vigorous intensity exercise minutes per week.

16) For each group, number of participants (denominator) included in each analysis and whether the analysis was by original assigned groups

### 16-i) Report multiple “denominators” and provide definitions

Report multiple “denominators” and provide definitions: Report N’s (and effect sizes) “across a range of study participation [and use] thresholds” [1], e.g., N exposed, N consented, N used more than x times, N used more than y weeks, N participants “used” the intervention/comparator at specific pre-defined time points of interest (in absolute and relative numbers per group). Always clearly define “use” of the intervention.

| | 1 | 2 | 3 | 4 | 5 | |
|---|---|---|---|---|---|---|
| subitem not at all important | ○ | ○ | ○ | ○ | ◉ | essential |

Clear selection

Does your paper address subitem 16-i? *

Copy and paste relevant sections from the manuscript (include quotes in quotation marks "like this" to indicate direct quotes from your manuscript), or elaborate on this item by providing additional information not in the ms, or briefly explain why the item is not applicable/relevant for your study

Participant flow through the study is presented in Figure 2. Of the 41 women who responded to the invitation, 22 were eligible and 21 consented to be randomized into either the combined exercise group (aerobic + resistance training; n=10) or the active control group (balance and flexibility; n=11), representing a 51.2% enrolment rate. Participants were divided into two cohorts: the first cohort (n=11) began in May 2023, and the second cohort (n=10) began in July 2023. The sample size aligns with previous research on feasibility and group cohesion for counselling programs for BCS [56,57]. Overall, 18 participants completed the 8-week intervention. Three participants withdrew after randomization, but before starting the intervention for reasons including a change in BC treatment plan (n=1), an issue with the timing of the programming and their schedule (n=1), and a change of interest in participating (n=1). An intention-to-treat analysis was conducted and therefore adherence was calculated based on the attendance and included the dropouts (n=3), since they were enrolled and randomized at the start of the intervention. Consequently, the attrition rate was 14.3%. Consequently, the attrition rate was 14.3%. For the ANCOVA analyses, complete case analysis was applied (n=18).

Attendance adherence rate for the remotely-delivered, combined exercise classes was 70.8% (17/24 classes) and 77.5% (3/4 sessions) for behavioral counselling sessions based on the M-PAC that were delivered only to those in the combined exercise group (n=10). Attendance adherence for the active control group was 70.8% (17/24 classes). Participants in the combined exercise group who completed the intervention and post-intervention measures (n=9) reported an average RPE of 12.8 and a HR of 111.8 during the resistance training classes, which corresponds to a moderate-intensity RPE and a HR within the light-moderate target heart rate zone. In addition, participants in the combined exercise intervention (n=9) reported a mean RPE of 12.2 and a mean HR of 119.7 during the unsupervised aerobic (i.e., walking) sessions, which corresponds moderate-intensity RPE and a HR within the moderate-intensity target heart rate zone. Participants in the active control group who completed the intervention and post-intervention measures (i.e., including the exercise log) (n=8) reported a mean RPE of 11.1 and a mean HR of 85.5 during their balance and flexibility program, which corresponds to a light-moderate intensity RPE and light-intensity target heart rate zone. There were no adverse events during the intervention in either group. The measurement completion rates for the study were as follows: 85.7% (18/21) for the participant satisfaction questionnaire and 81.0% (17/21) for the Working Alliance Inventory. For objectively measured cognitive function, completion rates were 85.7% (18/21) for the memory test and 81.0% (17/21) for the executive function test. Additionally, accelerometer wear compliance was achieved by 66.7% (14/21) of participants.

### 16-ii) Primary analysis should be intent-to-treat

Primary analysis should be intent-to-treat, secondary analyses could include comparing only "users", with the appropriate caveats that this is no longer a randomized sample (see 18-i).

| | 1 | 2 | 3 | 4 | 5 | |
|---|---|---|---|---|---|---|
| subitem not at all important | ○ | ○ | ○ | ◉ | ○ | essential |

Clear selection

### Does your paper address subitem 16-ii?

Copy and paste relevant sections from the manuscript (include quotes in quotation marks "like this" to indicate direct quotes from your manuscript), or elaborate on this item by providing additional information not in the ms, or briefly explain why the item is not applicable/relevant for your study

Participant flow through the study is presented in Figure 2. Of the 41 women who responded to the invitation, 22 were eligible and 21 consented to be randomized into either the combined exercise group (aerobic + resistance training; n=10) or the active control group (balance and flexibility; n=11), representing a 51.2% enrolment rate. Participants were divided into two cohorts: the first cohort (n=11) began in May 2023, and the second cohort (n=10) began in July 2023. The sample size aligns with previous research on feasibility and group cohesion for counselling programs for BCS [56,57]. Overall, 18 participants completed the 8-week intervention. Three participants withdrew after randomization, but before starting the intervention for reasons including a change in BC treatment plan (n=1), an issue with the timing of the programming and their schedule (n=1), and a change of interest in participating (n=1). An intention-to-treat analysis was conducted and therefore adherence was calculated based on the attendance and included the dropouts (n=3), since they were enrolled and randomized at the start of the intervention. Consequently, the attrition rate was 14.3%. Consequently, the attrition rate was 14.3%. For the ANCOVA analyses, complete case analysis was applied (n=18).

### 17a) For each primary and secondary outcome, results for each group, and the estimated effect size and its precision (such as 95% confidence interval)

Does your paper address CONSORT subitem 17a? *

Copy and paste relevant sections from the manuscript (include quotes in quotation marks "like this" to indicate direct quotes from your manuscript), or elaborate on this item by providing additional information not in the ms, or briefly explain why the item is not applicable/relevant for your study

Attendance adherence rate for the remotely-delivered, combined exercise classes was 70.8% (17/24 classes) and 77.5% (3/4 sessions) for behavioral counselling sessions based on the M-PAC that were delivered only to those in the combined exercise group (n=10). Attendance adherence for the active control group was 70.8% (17/24 classes). Participants in the combined exercise group who completed the intervention and post-intervention measures (n=9) reported an average RPE of 12.8 and a HR of 111.8 during the resistance training classes, which corresponds to a moderate-intensity RPE and a HR within the light-moderate target heart rate zone. In addition, participants in the combined exercise intervention (n=9) reported a mean RPE of 12.2 and a mean HR of 119.7 during the unsupervised aerobic (i.e., walking) sessions, which corresponds moderate-intensity RPE and a HR within the moderate-intensity target heart rate zone. Participants in the active control group who completed the intervention and post-intervention measures (i.e., including the exercise log) (n=8) reported a mean RPE of 11.1 and a mean HR of 85.5 during their balance and flexibility program, which corresponds to a light-moderate intensity RPE and light-intensity target heart rate zone. There were no adverse events during the intervention in either group. The measurement completion rates for the study were as follows: 85.7% (18/21) for the participant satisfaction questionnaire and 81.0% (17/21) for the Working Alliance Inventory. For objectively measured cognitive function, completion rates were 85.7% (18/21) for the memory test and 81.0% (17/21) for the executive function test. Additionally, accelerometer wear compliance was achieved by 66.7% (14/21) of participants.

Participant responses regarding program satisfaction are summarized in Table 3. Overall, participants reported high satisfaction with the intervention. The majority of participants (94.4%) agreed or strongly agreed that the program was rewarding and appreciated the virtual delivery format. Most participants (83.3%) agreed or strongly agreed that the intervention helped them increase their PA levels, and 77.7% indicated that they would recommend the program to other BC patients. Most participants found the intervention beneficial and participants (62.5%) agreed or strongly agreed that the behavioral counselling sessions were personally useful. Similarly, 61.1% of participants agreed or strongly agreed that the intervention was not burdensome.
Participant responses on the Working Alliance Inventory-Short Revised (WAI-SR) are summarized in Table 4. Overall, participants reported positive perceptions of their alliance with the QEP, with 88.2% very often or always feeling mutual respect and 76.5% feeling appreciated and cared for.

Changes in objectively measured cognitive function scores from baseline to post-intervention for the combined exercise group versus active control group are presented in Table 5. There were no statistical differences in objectively-measured cognitive function in the combined exercise group compared to the active control group. However, adjusted group mean differences favoring the combined exercise group were observed in the scores for the Picture Sequence Memory Task (mean change: +5.33, $\eta p^2=0.03$), List Sorting Working Memory Test (mean change: +8.17, $\eta p^2=0.12$), and Auditory Verbal Learning (mean change: +3.22, $\eta p^2=0.12$). Adjusted group mean differences favoring the combined exercise group were observed in the scores for the Picture Sequence Memory Task (mean change: +5.33, $\eta p^2=0.03$), List Sorting Working Memory Test (mean change: +8.17, $\eta p^2=0.12$), and Auditory Verbal Learning (mean change: +3.22, $\eta p^2=0.12$). On the other hand, adjusted group mean differences favored the active control group in the scores for the Oral Reading [illegible]

Recognition Test (mean changes: -9.65, $\eta p^2=0.14$) and Picture Vocabulary (mean changes: -2.48, $\eta p^2=0.20$). Table 5 also shows the four cognition outcomes from the PsyToolkit: Repeat trial time (ms), Switch trial time (ms), Repeat trials correct (%), Switch trials correct (%). No significant differences were found between the combined exercise group compared to the active control group. However, aA reduction in Repeat trial time favored the combined exercise group (mean change: -146.14 ms, $\eta p^2=0.04$). Likewise, a greater decrease in the switch trial time favored the combined exercise group (mean change: -394.35 ms, $\eta p^2=0.07$) compared to the control group (mean change: -124.39 ms, $\eta p^2=0.07$). For accuracy, improvements in Repeat trials correct (mean change: +4.86%, $\eta p^2=0.02$) and Switch trials correct (mean change: +19.70%, $\eta p^2=0.02$) also favored the combined exercise group, while the control group exhibited decreases in these measures.

Table 6 presents the changes in device-based exercise levels among BC patients. There were no statistical differences between the two groups. However, an increase in total moderate-to-vigorous intensity exercise minutes was observed from pre- to post-intervention, favoring participants in the active control group compared to those in the combined exercise group (mean difference in change = 58.8 minutes, $\eta p^2=0.06$).

## 17a-i) Presentation of process outcomes such as metrics of use and intensity of use

In addition to primary/secondary (clinical) outcomes, the presentation of process outcomes such as metrics of use and intensity of use (dose, exposure) and their operational definitions is critical. This does not only refer to metrics of attrition (13-b) (often a binary variable), but also to more continuous exposure metrics such as “average session length”. These must be accompanied by a technical description how a metric like a “session” is defined (e.g., timeout after idle time) [1] (report under item 6a).

| | 1 | 2 | 3 | 4 | 5 | |
|---|---|---|---|---|---|---|
| subitem not at all important | ○ | ○ | ○ | ◉ | ○ | essential |

Clear selection

Does your paper address subitem 17a-i?

Copy and paste relevant sections from the manuscript (include quotes in quotation marks "like this" to indicate direct quotes from your manuscript), or elaborate on this item by providing additional information not in the ms, or briefly explain why the item is not applicable/relevant for your study

Attendance adherence rate for the remotely-delivered, combined exercise classes was 70.8% (17/24 classes) and 77.5% (3/4 sessions) for behavioral counselling sessions based on the M-PAC that were delivered only to those in the combined exercise group (n=10). Attendance adherence for the active control group was 70.8% (17/24 classes). Participants in the combined exercise group who completed the intervention and post-intervention measures (n=9) reported an average RPE of 12.8 and a HR of 111.8 during the resistance training classes, which corresponds to a moderate-intensity RPE and a HR within the light-moderate target heart rate zone. In addition, participants in the combined exercise intervention (n=9) reported a mean RPE of 12.2 and a mean HR of 119.7 during the unsupervised aerobic (i.e., walking) sessions, which corresponds moderate-intensity RPE and a HR within the moderate-intensity target heart rate zone. Participants in the active control group who completed the intervention and post-intervention measures (i.e., including the exercise log) (n=8) reported a mean RPE of 11.1 and a mean HR of 85.5 during their balance and flexibility program, which corresponds to a light-moderate intensity RPE and light-intensity target heart rate zone. There were no adverse events during the intervention in either group. The measurement completion rates for the study were as follows: 85.7% (18/21) for the participant satisfaction questionnaire and 81.0% (17/21) for the Working Alliance Inventory. For objectively measured cognitive function, completion rates were 85.7% (18/21) for the memory test and 81.0% (17/21) for the executive function test. Additionally, accelerometer wear compliance was achieved by 66.7% (14/21) of participants.

17b) For binary outcomes, presentation of both absolute and relative effect sizes is recommended

Does your paper address CONSORT subitem 17b? *

Copy and paste relevant sections from the manuscript (include quotes in quotation marks "like this" to indicate direct quotes from your manuscript), or elaborate on this item by providing additional information not in the ms, or briefly explain why the item is not applicable/relevant for your study

See Table 5 and 6 for results

18) Results of any other analyses performed, including subgroup analyses and adjusted analyses, distinguishing pre-specified from exploratory

Does your paper address CONSORT subitem 18? *

Copy and paste relevant sections from the manuscript (include quotes in quotation marks "like this" to indicate direct quotes from your manuscript), or elaborate on this item by providing additional information not in the ms, or briefly explain why the item is not applicable/relevant for your study

See Table 5 and 6 for results

18-i) Subgroup analysis of comparing only users

A subgroup analysis of comparing only users is not uncommon in ehealth trials, but if done, it must be stressed that this is a self-selected sample and no longer an unbiased sample from a randomized trial (see 16-iii).

| | 1 | 2 | 3 | 4 | 5 | |
|---|---|---|---|---|---|---|
| subitem not at all important | ○ | ○ | ○ | ◉ | ○ | essential |

Clear selection

Does your paper address subitem 18-i?

Copy and paste relevant sections from the manuscript (include quotes in quotation marks "like this" to indicate direct quotes from your manuscript), or elaborate on this item by providing additional information not in the ms, or briefly explain why the item is not applicable/relevant for your study

not applicable and therefore not included

19) All important harms or unintended effects in each group
(for specific guidance see CONSORT for harms)

Does your paper address CONSORT subitem 19? *

Copy and paste relevant sections from the manuscript (include quotes in quotation marks "like this" to indicate direct quotes from your manuscript), or elaborate on this item by providing additional information not in the ms, or briefly explain why the item is not applicable/relevant for your study

no adverse events reported

19-i) Include privacy breaches, technical problems

Include privacy breaches, technical problems. This does not only include physical "harm" to participants, but also incidents such as perceived or real privacy breaches [1], technical problems, and other unexpected/unintended incidents. "Unintended effects" also includes unintended positive effects [2].

| | 1 | 2 | 3 | 4 | 5 | |
|---|---|---|---|---|---|---|
| subitem not at all important | ○ | ○ | ○ | ◉ | ○ | essential |

Clear selection

Does your paper address subitem 19-i?

Copy and paste relevant sections from the manuscript (include quotes in quotation marks "like this" to indicate direct quotes from your manuscript), or elaborate on this item by providing additional information not in the ms, or briefly explain why the item is not applicable/relevant for your study

no privacy breaches, technical problems.

### 19-ii) Include qualitative feedback from participants or observations from staff/researchers

Include qualitative feedback from participants or observations from staff/researchers, if available, on strengths and shortcomings of the application, especially if they point to unintended/unexpected effects or uses. This includes (if available) reasons for why people did or did not use the application as intended by the developers.

| | 1 | 2 | 3 | 4 | 5 | |
|---|---|---|---|---|---|---|
| subitem not at all important | ○ | ○ | ○ | ◉ | ○ | essential |

Clear selection

### Does your paper address subitem 19-ii?

Copy and paste relevant sections from the manuscript (include quotes in quotation marks "like this" to indicate direct quotes from your manuscript), or elaborate on this item by providing additional information not in the ms, or briefly explain why the item is not applicable/relevant for your study

not applicable therefore not included

## DISCUSSION

### 22) Interpretation consistent with results, balancing benefits and harms, and considering other relevant evidence

NPT: In addition, take into account the choice of the comparator, lack of or partial blinding, and unequal expertise of care providers or centers in each group

22-i) Restate study questions and summarize the answers suggested by the data, starting with primary outcomes and process outcomes (use)

Restate study questions and summarize the answers suggested by the data, starting with primary outcomes and process outcomes (use).

| | 1 | 2 | 3 | 4 | 5 | |
|---|---|---|---|---|---|---|
| subitem not at all important | ○ | ○ | ○ | ○ | ◉ | essential |

Clear selection

Does your paper address subitem 22-i? *

Copy and paste relevant sections from the manuscript (include quotes in quotation marks "like this" to indicate direct quotes from your manuscript), or elaborate on this item by providing additional information not in the ms, or briefly explain why the item is not applicable/relevant for your study

To our knowledge, this is the first study to pilot a remotely-delivered, supervised combined exercise program for BC patients following chemotherapy, exhibiting mild cognitive impairment.

22-ii) Highlight unanswered new questions, suggest future research

Highlight unanswered new questions, suggest future research.

| | 1 | 2 | 3 | 4 | 5 | |
|---|---|---|---|---|---|---|
| subitem not at all important | ○ | ○ | ○ | ○ | ◉ | essential |

Clear selection

Does your paper address subitem 22-ii?

Copy and paste relevant sections from the manuscript (include quotes in quotation marks "like this" to indicate direct quotes from your manuscript), or elaborate on this item by providing additional information not in the ms, or briefly explain why the item is not applicable/relevant for your study

There is a need for rigorous and well-designed RCTs. While the use of shorter exercise protocols like our study provides preliminary evidence for cognitive benefits, long-term exercise is more efficacious in addressing age-related cognitive decline and improving cognition. Future trials should include active control conditions, test multicomponent exercises (i.e., aerobic and resistance training), and examine mechanisms between the relationship of exercise and cognitive function. Given that CRCI is an emerging cancer survivorship issue, the next step for this work is to conduct a larger RCT that is adequately powered to examine the effects of a remotely-delivered, combined exercise intervention on cognitive function in BC patients following chemotherapy. If replicated, these findings have the potential to identify effective and modifiable management strategies to enhance cognitive health in BC patients.

20) Trial limitations, addressing sources of potential bias, imprecision, and, if relevant, multiplicity of analyses

20-i) Typical limitations in ehealth trials

Typical limitations in ehealth trials: Participants in ehealth trials are rarely blinded. Ehealth trials often look at a multiplicity of outcomes, increasing risk for a Type I error. Discuss biases due to non-use of the intervention/usability issues, biases through informed consent procedures, unexpected events.

| | 1 | 2 | 3 | 4 | 5 | |
|---|---|---|---|---|---|---|
| subitem not at all important | ○ | ○ | ○ | ○ | ◉ | essential |

Clear selection

Does your paper address subitem 20-i? *

Copy and paste relevant sections from the manuscript (include quotes in quotation marks "like this" to indicate direct quotes from your manuscript), or elaborate on this item by providing additional information not in the ms, or briefly explain why the item is not applicable/relevant for your study

Our trial should be interpreted within the context of important strengths and limitations. The remotely-delivered component of the trial was a strength as it removed pre-existing barriers to exercise (e.g., time constraints, access, hesitancy toward public space). The combined exercise and active control group sessions were delivered by knowledgeable QEPs who were engaging and provided modifications that allowed all BC patients to participate in exercise, despite any physical limitations. The addition of a recorded session reduced participant burden by providing provided flexibility and autonomy. Our study was strengthened by the inclusion of behavioral counselling sessions which were guided by the M-PAC framework to encourage exercise adoption and adherence [39]. The inclusion of a rigorous comparison group was an additional strength since both groups received supervised, remotely-delivered exercise programs at differing intensities. Finally, the use of a standardized, well-validated battery of cognitive tests via the NIH Toolbox was another strength and in line with the ICCTF's recommendations [6].

This study had a small sample size and heterogeneous sample which limits the generalizability of the findings, this feasibility study is crucial for calculating sample sizes and refine methodologies for larger, future trials. In addition, the sample was predominantly White, highly-educated, middle to older aged, and were within the early cancer survivorship period. The focus on early-stage BC patients within 48 months of completing treatment may not be generalizable to other cancer populations such as individuals with metastatic disease, or patients who are pretreatment, in treatment, or immediately post-treatment. Furthermore, the heterogeneity in the time since treatment in our study may preclude identifying the optimal exercise timing to prevent or ameliorate CRCI, which requires future investigations.

It is important to note that BC patients may be more likely to seek out care for cancer rehabilitation, and view themselves as a researcher participant, compared to BC patients who are more culturally and socially diverse populations. Future research should consider the recommendations for increasing racial and ethnic diversity in cancer clinical trials by the American Society of Clinical Oncology and Association of Community Cancer Centers Joint Research Statement [65]. Another limitation of this study was not accounting for menopausal status which could be a confounding variable that may impact cognitive function in BC patients. Future studies should consider chemotherapy-induced menopause as possible mechanisms for CRCI. Furthermore, our study did not include self-reported cognitive function which is another important indicator of CRCI as it taps into day-to-day cognitive performance in relation to cancer and its treatments [6, 68]. Therefore, including both self-report and objective cognitive function assessments in future research is warranted.

## 21) Generalisability (external validity, applicability) of the trial findings

NPT: External validity of the trial findings according to the intervention, comparators, patients, and care providers or centers involved in the trial

## 21-i) Generalizability to other populations

Generalizability to other populations: In particular, discuss generalizability to a general Internet population, outside of a RCT setting, and general patient population, including applicability of the study results for other organizations

| | 1 | 2 | 3 | 4 | 5 | |
|---|---|---|---|---|---|---|
| subitem not at all important | ○ | ○ | ○ | ◉ | ○ | essential |

Clear selection

## Does your paper address subitem 21-i?

Copy and paste relevant sections from the manuscript (include quotes in quotation marks "like this" to indicate direct quotes from your manuscript), or elaborate on this item by providing additional information not in the ms, or briefly explain why the item is not applicable/relevant for your study

This study had a small sample size and heterogeneous sample which limits the generalizability of the findings, this feasibility study is crucial for calculating sample sizes and refine methodologies for larger, future trials. In addition, the sample was predominantly White, highly-educated, middle to older aged, and were within the early cancer survivorship period. The focus on early-stage BC patients within 48 months of completing treatment may not be generalizable to other cancer populations such as individuals with metastatic disease, or patients who are pretreatment, in treatment, or immediately post-treatment. Furthermore, the heterogeneity in the time since treatment in our study may preclude identifying the optimal exercise timing to prevent or ameliorate CRCI, which requires future investigations.

21-ii) Discuss if there were elements in the RCT that would be different in a routine application setting

Discuss if there were elements in the RCT that would be different in a routine application setting (e.g., prompts/reminders, more human involvement, training sessions or other co-interventions) and what impact the omission of these elements could have on use, adoption, or outcomes if the intervention is applied outside of a RCT setting.

| | 1 | 2 | 3 | 4 | 5 | |
|---|---|---|---|---|---|---|
| subitem not at all important | ○ | ○ | ○ | ◉ | ○ | essential |

Clear selection

Does your paper address subitem 21-ii?

Copy and paste relevant sections from the manuscript (include quotes in quotation marks "like this" to indicate direct quotes from your manuscript), or elaborate on this item by providing additional information not in the ms, or briefly explain why the item is not applicable/relevant for your study

Overall, BC patients were highly satisfied with the intervention. Most BC patients found the study rewarding, enjoyed the virtual delivery format., would recommend the study to other BC patients, and did not find the study assessments burdensome. BC patients also felt a mutual respect with their QEP and felt appreciated by their QEP, further contributing to their positive experience. This suggests that few adjustments need to be made to the intervention or the study design before implementing a future RCT. In our study, an additional QEP attended the classes to monitor participant safety and to assist the lead QEP in managing the virtual environment. This was beneficial to both the lead QEP and BCS and future remotely-delivered interventions should consider a second QEP. Additionally, the QEP's personality, approachability, and teaching style played an important role in enjoyability and feasibility for BC patients. Future studies should consider QEPs with cancer-specific training to offer exercise modifications to BC patients with limitations/injuries. Finally, our classes included 15 minutes of optional time before or after each class for BC patients to interact for overall class enjoyment, accountability, and to help foster a sense of community. This was beneficial to the overall satisfaction of the trial and should be implemented in future studies.

OTHER INFORMATION

23) Registration number and name of trial registry

Does your paper address CONSORT subitem 23? *

Copy and paste relevant sections from the manuscript (include quotes in quotation marks "like this" to indicate direct quotes from your manuscript), or elaborate on this item by providing additional information not in the ms, or briefly explain why the item is not applicable/relevant for your study

The study is registered with http://ClinicalTrials.gov (ID NCT05704855)

24) Where the full trial protocol can be accessed, if available

Does your paper address CONSORT subitem 24? *

Cite a Multimedia Appendix, other reference, or copy and paste relevant sections from the manuscript (include quotes in quotation marks "like this" to indicate direct quotes from your manuscript), or elaborate on this item by providing additional information not in the ms, or briefly explain why the item is not applicable/relevant for your study

The study is registered with http://ClinicalTrials.gov (ID NCT05704855)

25) Sources of funding and other support (such as supply of drugs), role of funders

Does your paper address CONSORT subitem 25? *

Copy and paste relevant sections from the manuscript (include quotes in quotation marks "like this" to indicate direct quotes from your manuscript), or elaborate on this item by providing additional information not in the ms, or briefly explain why the item is not applicable/relevant for your study

Internal grant through the Faculty of Kinesiology and Physical Education at the University of Toronto

X27) Conflicts of Interest (not a CONSORT item)

X27-i) State the relation of the study team towards the system being evaluated

In addition to the usual declaration of interests (financial or otherwise), also state the relation of the study team towards the system being evaluated, i.e., state if the authors/evaluators are distinct from or identical with the developers/sponsors of the intervention.

| | 1 | 2 | 3 | 4 | 5 | |
|---|---|---|---|---|---|---|
| subitem not at all important | ○ | ○ | ○ | ◉ | ○ | essential |

Clear selection

Does your paper address subitem X27-i?

Copy and paste relevant sections from the manuscript (include quotes in quotation marks "like this" to indicate direct quotes from your manuscript), or elaborate on this item by providing additional information not in the ms, or briefly explain why the item is not applicable/relevant for your study

no COI to declare. Authors/evaluators are distinct from with the developers/sponsors of the intervention

About the CONSORT EHEALTH checklist

As a result of using this checklist, did you make changes in your manuscript? *

- ○ yes, major changes
- ◉ yes, minor changes
- ○ no

What were the most important changes you made as a result of using this checklist?

following standardized guidelines for reporting of e-health interventions

How much time did you spend on going through the checklist INCLUDING making changes in your manuscript *

1 week for changes to manuscript and checklist

As a result of using this checklist, do you think your manuscript has improved? *

- (x) yes
- ( ) no
- ( ) Other:

Would you like to become involved in the CONSORT EHEALTH group?

This would involve for example becoming involved in participating in a workshop and writing an "Explanation and Elaboration" document

- ( ) yes
- (x) no
- ( ) Other:

Clear selection

Any other comments or questions on CONSORT EHEALTH

not applicable and therefore no comments

STOP - Save this form as PDF before you click submit

To generate a record that you filled in this form, we recommend to generate a PDF of this page (on a Mac, simply select "print" and then select "print as PDF") before you submit it.

When you submit your (revised) paper to JMIR, please upload the PDF as supplementary file.

Don't worry if some text in the textboxes is cut off, as we still have the complete information in our database. Thank you!

Final step: Click submit !

Click submit so we have your answers in our database!

Submit Clear form

Never submit passwords through Google Forms.

This content is neither created nor endorsed by Google. - Terms of Service - Privacy Policy

Does this form look suspicious? Report

Google Forms
